# Interferon-stimulated gene 20 (ISG20) selectively degrades N6-methyladenosine modified Hepatitis B Virus transcripts

**DOI:** 10.1371/journal.ppat.1008338

**Published:** 2020-02-14

**Authors:** Hasan Imam, Geon-Woo Kim, Saiful Anam Mir, Mohsin Khan, Aleem Siddiqui

**Affiliations:** Division of Infectious Diseases, Department of Medicine, University of California, San Diego, La Jolla, California, United States of America; The Pennsylvania State University College of Medicine, UNITED STATES

## Abstract

Interferon (IFN) stimulates a whole repertoire of cellular genes, collectively referred to as ISGs (Interferon-stimulated genes). ISG20, a 3´-5´ exonuclease enzyme, has been previously shown to bind and degrade hepatitis B Virus (HBV) transcripts. Here, we show that the N6-methyladenosine (m^6^A)-modified HBV transcripts are selectively recognized and processed for degradation by ISG20. Moreover, this effect of ISG20 is critically regulated by m^6^A reader protein, YTHDF2 (YTH-domain family 2). Previously, we identified a unique m^6^A site within HBV transcripts and confirmed that methylation at nucleotide A1907 regulates HBV lifecycle. In this report, we now show that the methylation at A1907 is a critical regulator of IFN-α mediated decay of HBV RNA. We observed that the HBV RNAs become less sensitive to ISG20 mediated degradation when methyltransferase enzymes or m^6^A reader protein YTHDF2 are silenced in HBV expressing cells. By using an enzymatically inactive form ISG20^D94G^, we further demonstrated that ISG20 forms a complex with m^6^A modified HBV RNA and YTHDF2 protein. Due to terminal redundancy, HBV genomic nucleotide A1907 position is acquired twice by pregenomic RNA (pgRNA) during transcription and therefore the sites of methylation are encoded within 5´ and 3´ epsilon stem loops. We generated HBV mutants that lack m^6^A site at either one (5´ or 3´) or both the termini (5´& 3´). Using these mutants, we demonstrated that m^6^A modified HBV RNAs are subjected to ISG20-mediated decay and propose sequence of events, in which ISG20 binds with YTHDF2 and recognizes m^6^A-modified HBV transcripts to carry out the ribonuclease activity. This is the first study, which identifies a hitherto unknown role of m^6^A modification of RNA in IFN-α induced viral RNA degradation and proposes a new role of YTHDF2 protein as a cofactor required for IFN-α mediated viral RNA degradation.

## Author summary

Hepatitis B Virus (HBV) is a DNA virus but replicates through a transitional pregenomic RNA (pgRNA). Interferon stimulated antiviral RNase, ISG20 selectively binds to the lower epsilon stem loop of HBV RNA and causes their degradation. Surprisingly this ISG20 binding site is chemically modified by N6-methyladenosine addition to A1907 residue, which resides in the lower region of the epsilon stem loop. This single m^6^A site occurs twice due to terminal redundancy of sequences in the pgRNA. We demonstrated herein that IFN-α-induced ISG20 can selectively degrade m^6^A modified HBV RNA. Using a combined strategy of silencing cellular methyltransferases, m^6^A binding protein YTHDF2 and the m^6^A sites mutants, we show that HBV transcripts are resistant to either IFN-α treatment or ectopically introduced ISG20 mediated degradation. YTHDF2 is an m^6^A binding protein which makes the HBV RNAs less stable. YTHDF2 protein forms a complex with IFN-α stimulated ISG20 and executes the nuclease digestion of the recruited m^6^A modified transcripts. Absence of cellular m^6^A machinery (methyltransferases or m^6^A reader proteins) makes the HBV RNA unresponsive to ISG20 mediated decay. This study provides molecular explanation of IFN-α mediated degradation of m^6^A modified HBV RNAs.

## Introduction

IFNs are a family of secretory proteins with the ability to impede viral infection and replication [1–3]. Type 1 IFNs initiate a signaling cascade via IFN-α/β receptors (IFNAR) through the Jak-STAT (Janus Kinase-Signal Transducer and Activator of Transcription) pathway, which transcribes hundreds of IFN-stimulated genes [4, 5]. IFN-stimulated ISG20 is a 20-kDa protein, which has 3´ -5´ exonuclease activity and cleaves single-stranded RNA and DNA [6–9].

Methylation at the N6 position of adenosine (m^6^A) is the most abundant internal modification of cellular mRNAs, viral transcripts, microRNAs (miRNAs) and long noncoding RNAs (lncRNAs) in eukaryotic cells, which modulates RNA structure, function and localization [10, 11]. A multicomponent methyltransferase complex containing the methyltransferase-like (METTL) enzymes METTL3 and METTL14 and the cofactors Wilms tumor 1-associated protein (WTAP) catalyzes m^6^A modification [10–14], which in turn is removed by demethylases fat mass and obesity-associated protein (FTO) and/or α-ketoglutarate-dependent dioxygenase AlkB homolog 5 (ALKBH5) [15, 16]. The cytoplasmic YTHDF1, YTHDF2, and YTHDF3 proteins bind with m^6^A modified RNA through their C-terminal YTH domain and therefore these proteins are known as m^6^A ‘readers’. Interestingly, m^6^A readers interact with the cellular exonucleases and permit m^6^A-containing RNAs to be degraded in the cytoplasm. For example, YTHDF1 protein promotes the translation of m^6^A-modified mRNA where YTHDF2 targets the m^6^A-modified mRNAs for degradation [17, 18]. Moreover, YTHDF2 can also recruit CCR4-NOT (C-C motif chemokine receptor 4—negative on TATA-less) deadenylase complex by directly interacting with the SH-domain of CNOT1 (CCR4-NOT Transcription Complex Subunit 1), the scaffolding subunit of the complex, to initiate deadenylation and decay of m^6^A-containing mRNAs [19]. Another m^6^A reader protein YTHDC2 plays an important role in regulating mRNA stability by mediating an interaction with the XRN1 (5´-3´exoribonuclease 1) [20]. Altogether, these previous observations establish that the m^6^A modification is intricately linked with the RNA degradation machinery.

HBV infection is one of the major causes of chronic hepatitis which is associated with elevated risk of severe liver diseases, fibrosis, cirrhosis, and primary hepatocellular carcinoma [21]. HBV contains a DNA genome but amplifies through a unique intermediate pgRNA by reverse transcription [22, 23]. The formation of covalently closed circular DNA (cccDNA) follows after a 3.2kb relaxed circular (rc) viral DNA genome has entered into the nucleus. cccDNA serves as a template for the production of five viral mRNAs (3.5~3.6 kb pre-core mRNA, 3.5 kb pre-genomic RNA, 2.4 and 2.1 kb surface antigen mRNA, and 0.7 kb X mRNA). They are transcribed from different initiation sites but terminated at a single polyA site. HBV pgRNA contains epsilon (ε) stem loop structure and serves as a template for reverse transcription and formation of viral core protein and polymerase protein [22, 23]. HBV infection does not induce IFN production but the virus programs infected cells to effectively suppress its synthesis [23, 24]. IFN-α treatment inhibits HBV replication by purging pgRNA/capsid formation, by affecting RNA transcription from the cccDNA in an epigenetic manner, events that ultimately lead to blocking further steps of viral replication [25–27]. IFN treatment of HBV patients only affects with ~30% efficacy [21].

Our previous study identified a single m^6^A consensus DRACH motif (GGACA) within the epsilon stem loop structure of all HBV RNAs which is repeated twice in the 5´ and 3´ ends of the pgRNA due to terminal redundancy but resides only once in the 3´ noncoding sequences of the subgenomic transcripts [28]. Liu et al. reported that IFN induces ISG20, which can bind to the epsilon stem loop structure of HBV RNA and inhibit viral replication via its exonuclease activity [29]. The ISG20 binding site was mapped at lower stem of epsilon loop, which overlaps with the unique m^6^A consensus motif (GGACA), we identified previously. We, therefore, investigated if methylation status of this consensus plays any role in ISG20 binding and subsequent degradation. In this study, we demonstrate that IFN-α induced ISG20 selectively degrades m^6^A containing HBV RNAs. Our work also shows that ISG20 and YTHDF2 form a complex, in which recruited ISG20 causes the degradation of HBV transcripts. Mutation in m^6^A sites abrogates ISG20 mediated RNA decay. This work highlights a new role of m^6^A modification in IFN-α mediated degradation of HBV RNA and provides a molecular explanation of the effects of IFN on HBV replication observed previously [27].

## Results

### m^6^A modified HBV transcripts are subject to degradation by IFN-α induced ISG20

**Fig 1:**
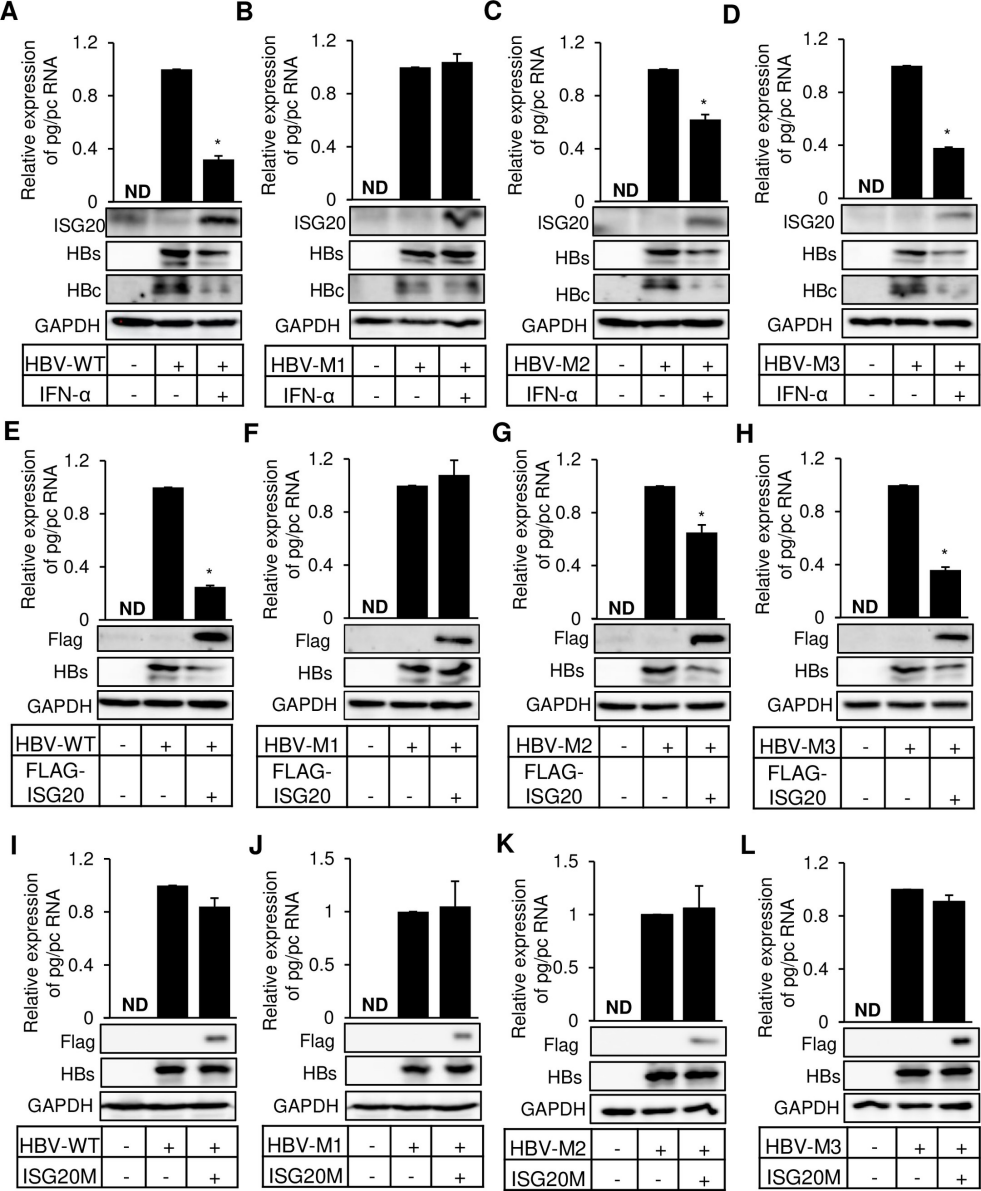
IFN-α induced ISG20 and ectopically expressed ISG20 can degrade m^6^A modified HBV RNA. **(A)** HBV-WT **(B)** HBV-M1 **(C)** HBV-M2 **(D)** HBV-M3 plasmids were transfected into HepG2 cells and incubated for 48h until harvest and IFN-α was added (2000 IU/ml) 24h before harvesting the cells. After RNA isolation relative expression of pg/pc RNA was analyzed through RT-qPCR. **(E)** HBV-WT **(F)** HBV-M1 **(G)** HBV-M2 **(H)** HBV-M3 plasmids were co-transfected with FLAG-ISG20 into HepG2 cells and incubated for 48h until harvest. After RNA isolation relative expression of pg/pc RNA was checked through RT-qPCR. **(I)** HBV-WT **(J)** HBV-M1 **(K)** HBV-M2 **(L)** HBV-M3 plasmids were co-transfected with FLAG-ISG20M (mutated FLAG-ISG20) into HepG2 cells and incubated for 48h before harvesting the cells. After RNA isolation relative expression of pg/pc RNA was checked through RT-qPCR. The data for this figure are from three independent experiments and the bars represent the mean ± SD. ND, not detected. *P ≤0.05 by unpaired Student’s *t* test.

IFN-α treatment of HBV infected hepatocytes effectively decreases HBV replication by targeting pgRNA, its packaging, and transcription [25–27]. Here, we asked the question whether epitranscriptomic modification of HBV RNAs regulates IFN-α mediated degradation scheme. We have previously identified a unique m^6^A-consensus motif GGA*CA (1905–1909 relative to the unique single EcoRI site), where A* residue is the site for N6-methyladenosine modification. Liu et al. identified a stretch of sequences as the target of ISG20 binding, which harbors the single m^6^A modified site (A1907) that leads to HBV RNA degradation [29]. We used previously generated various combinations of mutations of m^6^A (A1907C) modified site [28]. HBV transcripts synthesized by mutant M1 are defective in m^6^A modifications at both 5´ and 3´ termini, while the mutants M2 and M3 either lack m^6^A modification at 5´ or 3´ epsilon respectively (S1A Fig). We also used HBV-M1 expressing RNAs in a MeRIP RT-qPCR analysis and found that m^6^A specific antibody failed to capture any HBV RNA (S1B Fig). This result thus rules out the existence of any additional m^6^A site other than A1907 in HBV RNA. All the mutants, along with the wild type (HBV-WT) were transfected into HepG2 cells and treated with IFN-α. IFN-α treatment of HBV-WT expressing cells leads to decreased levels of HBV RNAs (Fig 1A), whereas IFN-α treatment showed no apparent decrease in the HBV RNA expressed by HBV-M1 (Fig 1B). Since the HBV RNA expressed by HBV-M1 lack m^6^A sites and are therefore resistant to the ISG20 mediated degradation. We next analyzed the effect of IFN-α on the HBV mutants in which this m^6^A modification was only present either at 5´ (HBV-M2) or 3´ end (HBV-M3). Interestingly both the mutants showed significant but relatively lower levels of decrease (Fig 1C and 1D). Since the effect of IFN-α treatment is primarily mediated by ISG20 upregulation, we co-transfected HepG2 cells with HBV and ISG20 expression vector. The results show that when ISG20 was ectopically expressed, the m^6^A mutants showed similar pattern of sensitivity (Fig 1E–1H) as seen in case of IFN-α treatment (Fig 1A–1D). These data together (Fig 1A–1H), revealed that the m^6^A modification of HBV RNA critically regulates the effect of IFN-α treatment, which is mediated by ISG20 upregulation. It is well accepted that the effect of ISG20 on HBV transcripts is exerted by its exonuclease activity and to confirm this, we next used the defective mutant of ISG20. D94G mutation in exonuclease domain of ISG20 makes it exonuclease defective. Ectopic expression of ISG20M (FLAG-ISG20^D94G^) resulted in no changes in the levels of HBV RNA (Fig 1I–1L), consistent with its defect in nuclease activity, reinforcing the notion that ISG20 targets m^6^A modified RNAs.

We analyzed HBV RNA and few known cellular RNAs (CREBBP, PTEN, ATG5, LC3B, HOTAIR and NRON) upon IFN-α treatment in HBV expressing cells and surprisingly found no changes in cellular RNA expression although CREBBP and PTEN mRNAs are known to be m^6^A modified (S1C Fig), clearly suggesting that these effects of IFN-α are only specific to viral transcripts. Collectively these results reveal that ISG20 mediated purging of HBV RNAs is regulated by m^6^A modification and IFN-α induced degradation machinery requires m^6^A methylation of HBV RNA.

### ISG20 and m^6^A reader protein YTHDF2 are interacting partners

**Fig 2:**
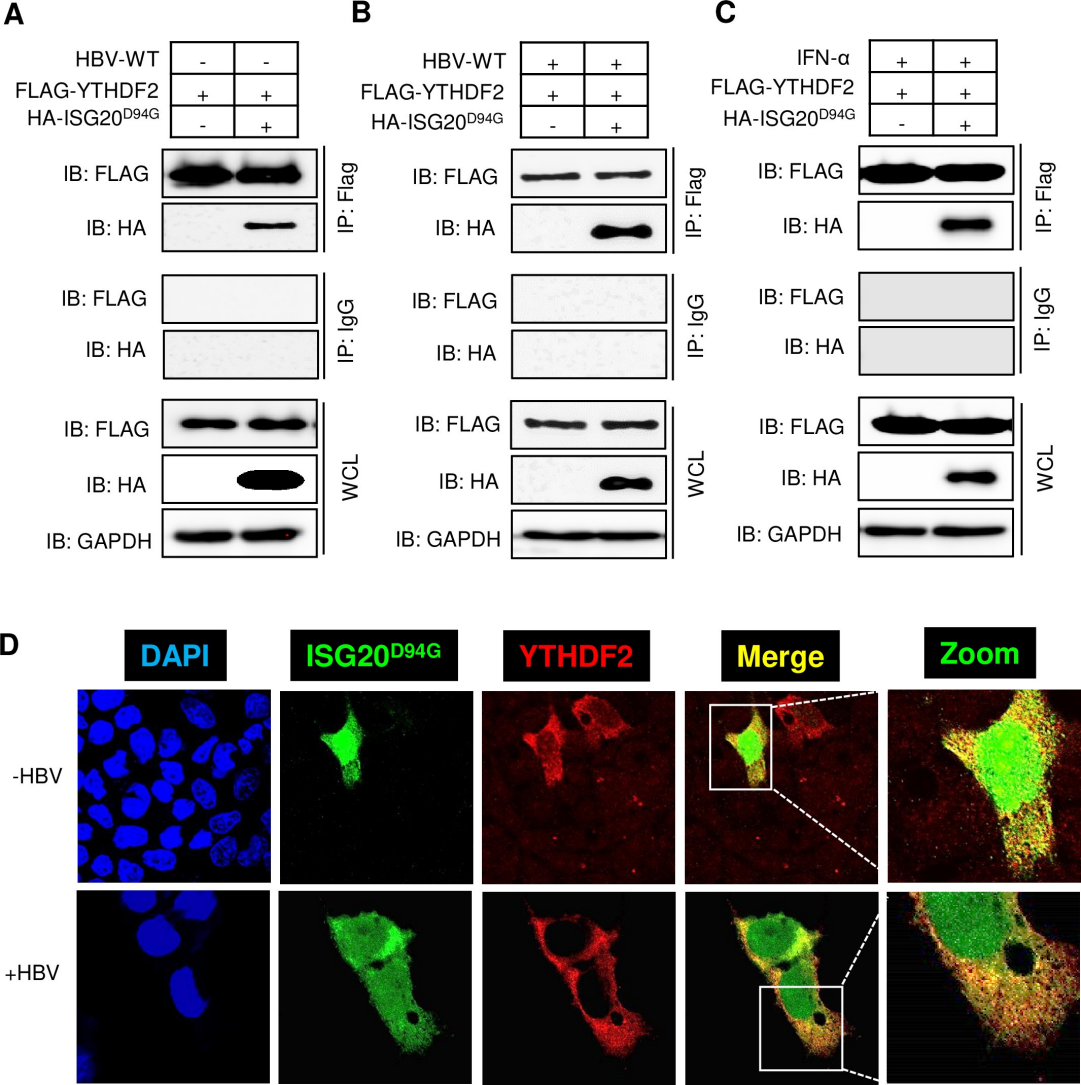
HBV-independent interaction between YTHDF2 proteins with ISG20. **(A)** FLAG-YTHDF2 and HA-ISG20^D94G^ plasmids were co-transfected into HepG2 cells. After 48h of incubation cells were harvested and lysates were prepared. FLAG antibody was used for IP and then probed with FLAG and HA antibody after Western Blot. **(B)** FLAG-YTHDF2 and HA-ISG20^D94G^ plasmids were co-transfected into HBV expressing HepG2 cells. After 48 hour of incubation cells were harvested and lysates were prepared. IP was done with FLAG antibody and then probed with FLAG and HA antibody after Western Blot. **(C)** FLAG-YTHDF2 and HA-ISG20^D94G^ plasmids were co-transfected into HepG2 cells and then incubated for 48h until harvest and IFN-α was added (2000 IU/ml) 24h before harvesting the cells. After preparing the lysates IP was done with FLAG antibody and probed with FLAG and HA antibody after Western Blot. **(D)** Confocal microscopy of HepG2 cells transfected with HA-ISG20^D94G^ and FLAG-YTHDF2, showing signals for DAPI stained nuclei (blue), ISG20^D94G^ (green), YTHDF2 (red) and the merged images (yellow) (upper panel). Confocal microscopy of HBV expressing HepG2 cells transfected with HA-ISG20^D94G^ and FLAG-YTHDF2, showing signals for DAPI stained nuclei (blue), ISG20^D94G^ (green), YTHDF2 (red) and the merged images (yellow) (lower panel). The data for this figure are from two independent experiments and the bars represent the mean ± SD.

m^6^A reader protein YTHDF2 is an m^6^A binding cellular protein, which regulate the stability of m^6^A modified RNAs. It is therefore conceivable that the YTHDF2 can bind to the lower stem of epsilon loop where the m^6^A site is situated. On the other hand, ISG20 also targets the same site of m^6^A modification. Since ISG20 and YTHDF2 proteins bind with HBV transcripts within close proximity, it is therefore likely that these proteins are interacting partners. To demonstrate interaction between these two proteins, we co-transfected FLAG-YTHDF2 and HA-ISG20^D94G^ plasmids (mutated ISG20 which can bind with HBV RNA but lacks exonuclease activity) into HepG2 cells with and without HBV-WT plasmid. Cellular lysates were immunoprecipitated using FLAG antibody which recognizes YTHDF2 and immunoblotted using an HA antibody which recognizes ISG20. Co-immunoprecipitation results shown in Fig 2A and Fig 2B clearly indicated that these proteins are held together in a complex. The results also demonstrate that YTHDF2 and ISG20 interaction is HBV-independent. We further confirmed that IFN-α treatment does not interrupt this protein complex formation if the HA-ISG20^D94G^ mutant was used in the analysis (Fig 2C). Using confocal microscopy, we confirmed the interaction between YTHDF2 and ISG20^D94G^, in which a merge of green (ISG20^D94G^) and red (YTHDF2) was evident (Fig 2D) to confirm their interaction. It should be noted that HBV gene expression caused an alteration in ISG20^D94G^ subcellular distribution from nucleus to cytoplasm, consistent with the degradation machinery being cytoplasmic.

### Interaction of YTHDF2 with ISG20 facilitates m^6^A modified HBV RNA degradation

**Fig 3:**
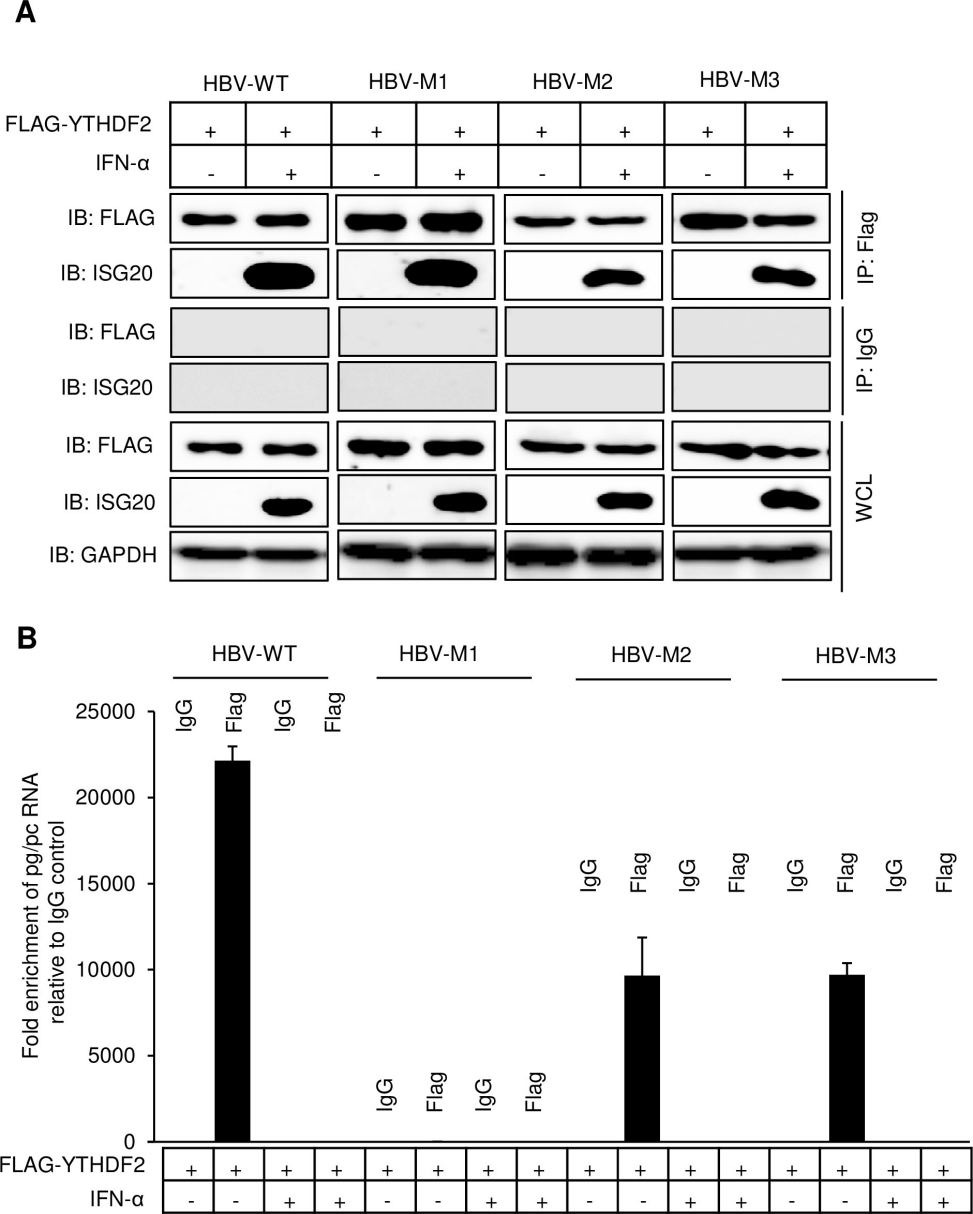
IFN-α induced ISG20 degrades m^6^A methylated HBV RNA *via* YTHDF2. **(A)** HBV-WT, HBV-M1, HBV-M2 and HBV-M3 plasmids were separately co-transfected with FLAG-YTHDF2 into HepG2 cells and incubated for 48h until harvest and IFN-α was added (2000 IU/ml) 24h before harvesting the cells. After preparing the lysates FLAG-IP was done and probed with FLAG and ISG20 antibody after Western Blot. **(B)** RNA was isolated from the final eluted products from co-IP experiment and checked for pg/pc RNA expression by RT-qPCR. The data for this figure are from two independent experiments and the bars represent the mean ± SD.

m^6^A modified HBV transcripts are bound by m^6^A reader YTHDF2 protein. To identify whether HBV RNA degradation is caused by YTHDF2-ISG20 interaction, we co-transfected HepG2 cells with FLAG-YTHDF2 along with either HBV-WT or mutants (HBV-M1, HBV-M2 and HBV-M3). Since this assay was aimed to examine the endogenous ISG20 levels, cells were treated with IFN-α (2000U/ml) and allowed 24h for induction of ISG20. Cellular lysates were immunoprecipitated using FLAG antibody followed by Western blot assays using ISG20 antibody. The results indicated that YTHDF2 and endogenous ISG20 interacted with each other and this interaction is m^6^A site-independent as the mutants also displayed similar interactions (Fig 3A). From the remaining co-IP eluted fraction, we isolated RNA and measured the HBV RNA expression level by RT-qPCR. In absence of IFN-α, elutes obtained from HBV-WT expressing cells showed significant HBV RNA levels but INF-α treated elutes showed a dramatic decrease in HBV RNA levels consistent with well-known effect of IFN-α. However, double mutant (HBV-M1) in which both 5´ and 3´ m^6^A sites are altered, there was no detectable HBV RNAs seen regardless of IFN-α treatment (Fig 3B). Whereas both HBV-M2 and HBV-M3, which contain m^6^A mutation in either 5´ or 3´ epsilon, HBV RNAs were present, although at moderate levels in the absence of IFN-α but not when IFN-α was present (Fig 3B). This level of IFN-α sensitivity is due to the fact that each epsilon with m^6^A modification may trigger partial degradation. These results clearly suggest that m^6^A modified RNAs are selectively degraded by IFN-α treatment which carries out this activity through the coordinated action of ISG20-YTHDF2 interaction.

In another analysis, HBV-WT or HBV mutants (HBV-M1/M2/M3) co-expressing YTHDF2 cellular lysates in the presence of IFN-α, were used to immunoprecipitate with ISG20 antibody followed by immunoblotting with FLAG antibody which can recognize YTHDF2. Similar to the result shown in Fig 3A, endogenous ISG20 and YTHDF2 were co-immunoprecipitated (S2A Fig) but HBV RNA-protein interaction pattern was different. No HBV RNA was observed from the RNAs isolated from final eluted products (S2B Fig), because RNAs from HBV-WT and M2-M3 were degraded in presence of IFN-α. Since HBV-M1 lacks m^6^A sites, they were not recognized by IFN-α-induced endogenous ISG20.

We next carried out similar studies using ISG20^D94G^ mutant which lacks the exonuclease activity but nevertheless binds YTHDF2. There was no IFN-α treatment in this analysis. Co-IP experiments using HBV-WT and M1-M3 plasmids showed results similar to those described above in Fig 3A (S2C Fig). First the interaction between YTHDF2 and ISG20^D94G^ occurred similar to wild type ISG20 (S2C Fig). But the HBV RNA pattern of expression, was slightly different. Since ISG20^D94G^ lacks exonuclease activity, there was no degradation of HBV transcripts. HBV-M1 again showed no evidence of HBV RNAs suggesting that the ISG20^D94G^ did not recognize non-m^6^A modified RNAs (S2D Fig). HBV-M2 and HBV-M3 showed modestly lower levels of HBV RNAs as either contained at least one m^6^A modification site.

### YTHDF2 facilitates IFN-α induced ISG20 degradation of m^6^A methylated HBV RNA

**Fig 4:**
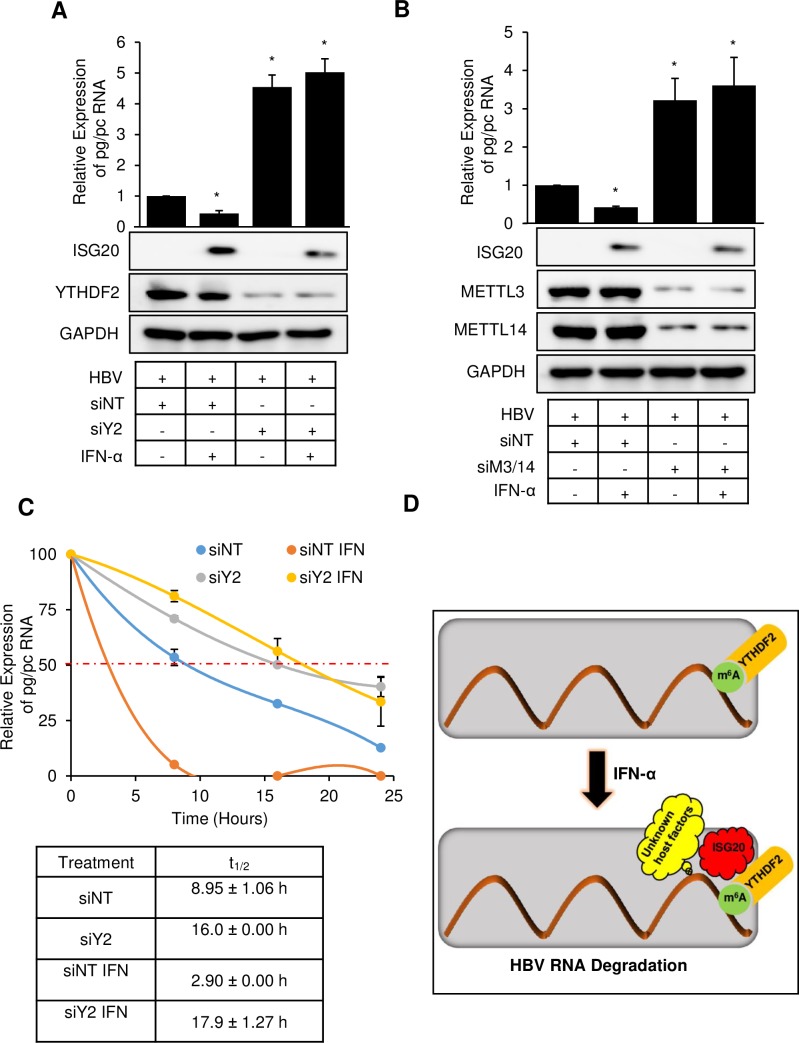
YTHDF2 protein facilitates the degradation of m^6^A methylated HBV RNA by IFN-α induced ISG20. **(A)** YTHDF2 was knocked down in HBV expressing HepG2 cells along with scrambled siRNA. IFN-α was added (2000 IU/ml) 24h before harvesting the cells. RNA was isolated from the cells and checked for pg/pc RNA expression using RT-qPCR. **(B)** METTL3/14 was knocked down in HBV expressing HepG2 cells along with scrambled siRNA. IFN-α was added (2000 IU/ml) 24h before harvesting the cells. RNA was isolated from the cells and checked for pg/pc RNA expression using RT-qPCR. **(C)** RT-qPCR analysis of pg/pc RNA relative to GAPDH in HBV-WT expressing HepG2 cells. The HBV-WT transfected HepG2 cells were depleted for YTHDF2 by specific siRNA, following Actinomycin D treatment at 24h post-siRNA transfection with and without IFN-α treatment. RNA was harvested at 0, 8, 16, and 24h post Actinomycin D treatment and relative levels of remaining HBV transcripts were analyzed. IFN-α was treated (2000 IU/ml) 24h before harvesting the cells. **(D)** Proposed model for ISG20 mediated degradation of m^6^A modified HBV RNA degradation. ISG20 exonuclease enzyme is released upon IFN-α treatment and is recruited by m^6^A reader protein YTHDF2 which facilitates m^6^A modified HBV RNA to degrade. The data for this figure are from two independent experiments and the bars represent the mean ± SD. *P ≤0.05 by unpaired Student’s *t* test.

We then asked whether cellular m^6^A machinery (both methyltransferases and m^6^A reader protein YTHDF2) plays a role in m^6^A modified HBV RNA degradation via IFN-α induced ISG20. To address this issue, we depleted YTHDF2 using specific siRNA in HBV expressing HepG2 cells and then treated with IFN-α. In control siRNA treated HBV expressing cells, IFN-α induced ISG20 downregulated the wild type HBV transcripts. However, the HBV RNA degradation was not observed in YTHDF2 depleted cells (Fig 4A). Similar results were obtained in IFN-α treated HBV expressing HepG2 cells depleted with methyltransferases METTL3/14 (Fig 4B). YTHDF2 cannot bind HBV RNA in methyltransferases-depleted cells, as the RNAs are not m^6^A modified and IFN-α-induced ISG20 cannot execute the exonuclease activity although it forms a complex with YTHDF2 protein. These data support the model in which both cellular methyltransferases METLL3 and METTL14 and m^6^A reader protein YTHDF2 are required for ISG20-mediated m^6^A modified HBV RNA degradation. Further, YTHDF2 protein facilitates the decay of m^6^A modified HBV RNA through IFN-α-induced exonuclease protein ISG20 by recruiting it.

We also determined the RNA stability of HBV transcripts in absence of YTHDF2 with IFN-α treatment. RNA stability analysis was carried out by Actinomycin D treatment and a time course of RNA degradation. This analysis (Fig 4C) demonstrated that IFN-α treatment causes total HBV RNA decay (orange), whereas YTHDF2 depleted cells, with or without IFN-α treatment renders HBV RNAs relatively more stable (yellow and gray). Control siRNA treated cells, without IFN-α treatment showed relatively stable profile (blue) (Fig 4C).

## Discussion

**Fig 5:**
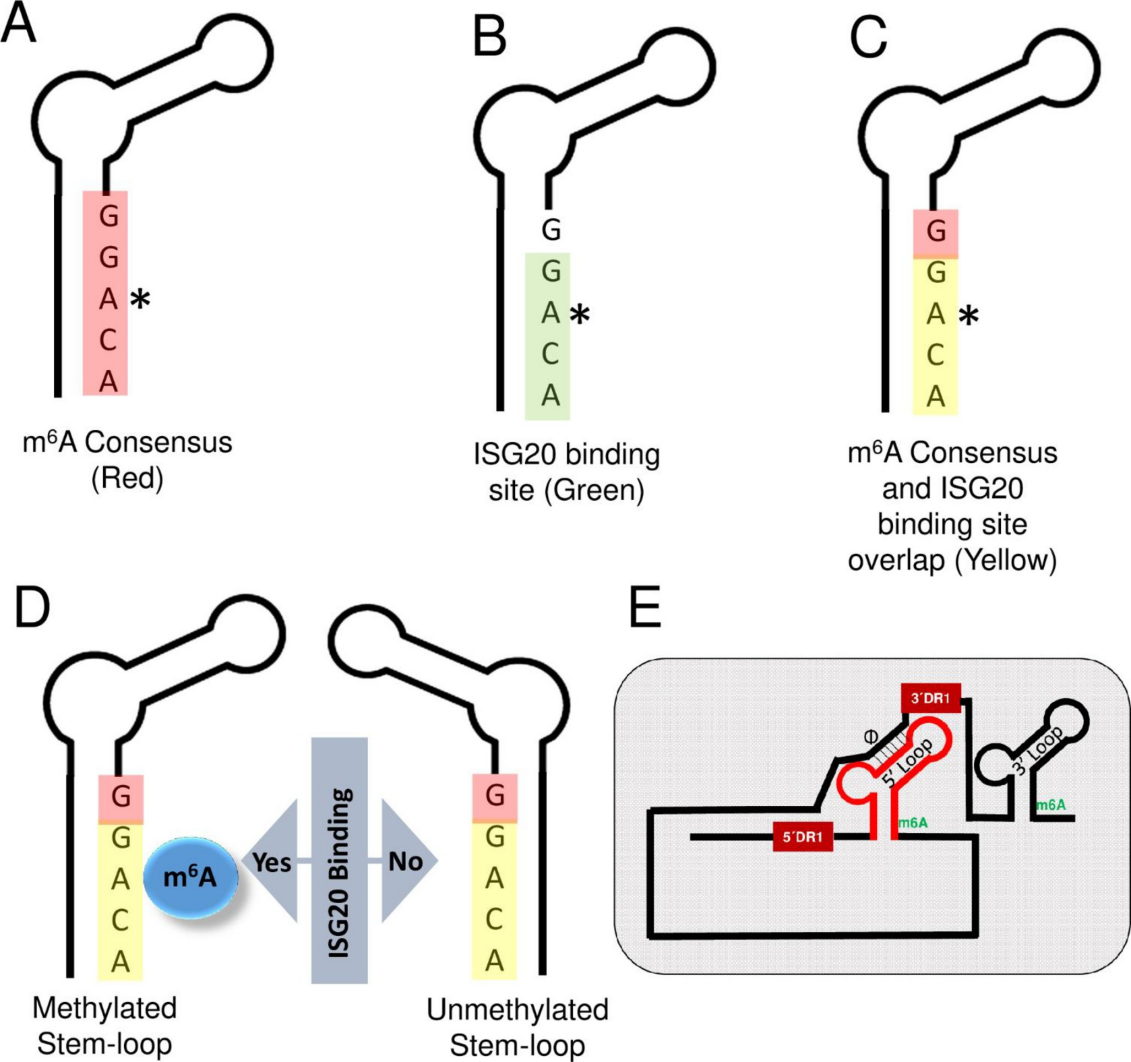
Schematics indicating the location of ISG20 binding site in the lower ε stem loop of HBV RNA. **(A)** m^6^A consensus motif (red) **(B)** ISG20 binding site (green) **(C)** m^6^A consensus and ISG20 binding site overlap (yellow) **(D)** ISG20 binding site in m^6^A methylated and unmethylated stem-loop of HBV RNA. **(E)** Proposed model for HBV long transcript for showing how cis-elements can juxtapose both the termini as described in discussion.

IFN-α induced ISG20 exonuclease has been reported to inhibit the replication of a number of viruses which include; HBV, Hepatitis C virus (HCV), West Nile virus, Dengue virus and Human Immunodeficiency virus (HIV) [29–31]. In the case of HCV replication, IFN effect is mediated by multiple antiviral pathways [30], while ISG20 overexpression in HIV expressing cells is associated with delayed HIV-1 replication [31]. IFN-induced upregulation of endogenous ISG20 inhibits HBV replication via viral RNA degradation [29]. ISG20 directly binds to the lower stem region of ε loop of HBV RNA (Fig 5B). Interestingly this ISG20 binding site contains an m^6^A consensus motif we have recently identified (Fig 5C) [28]. Since the RNA-protein interaction is one of the major events regulated by m^6^A modification, we sought to investigate if the methylation status of this ISG20 binding site can modulate ISG20- ε loop interaction (Fig 5D). The present analysis described here convincingly shows that ISG20- ε loop interaction is critically regulated by the m^6^A modification and impacts subsequent events of HBV RNA degradation. The mutational analysis of the DRACH motif further confirmed that only m^6^A modified HBV RNAs are targeted by ISG20 exonuclease, which eventually degrades all the viral transcripts including pgRNA (Fig 1). The mutant HBV-M1, which expresses HBV transcripts lacking m^6^A at both termini of pgRNA was resistant to IFN-α and ISG20-mediated suppression (Fig 1). Our work further adds to the mechanism(s) of IFN-induced antiviral programs including those of its effect on HBV transcription.

ISG20’s exonuclease activity is broadly nonspecific and has been found to degrade RNA genome of several RNA viruses [9, 29, 32]. In contrast, these investigations did not identify the exact mechanism(s) of viral RNA degradation by ISG20 [33, 34]. Liu et al., identified the ISG20 binding site in the lower stem of ε loop of viral transcripts [29]. Studies suggest that in order to become ISG20 sensitive, any viral RNA must possess unique binding site(s) to facilitate its targeting by ISG20. While our work suggests that the epitranscriptomic modification of viral RNA is targeted by ISG20, additional work is needed to characterize the sequence specificity and or local RNA structure which dictates ISG20-RNA interaction leading to enzymatic degradation. These studies will reveal the exact target structure of ISG20-mediated RNA decay process.

We noted that ISG20 mainly targeted viral RNA and analysis of a number of host RNAs were found to lack ISG20 sensitivity (S1C Fig). This observation supports the view that there is an inherent mechanism that determines the self/non-self-target specificity, leading to selective degradation of viral RNA. How ISG20 or IFN can target the RNA of foreign origin remains to be studied. However, our study sheds some light in this direction. Out data unambiguously points out that the m^6^A modification could be a possible marker that flags the target transcripts to be recognized by ISG20 for subsequent degradation. This is evidenced by the fact that despite the presence of ISG20 binding site in the HBV genome, the IFN-α treatment failed to degrade HBV transcripts when cellular methyltransferases were depleted (Fig 4B). This suggests that presence of an ISG20 binding site within the transcript may not be sufficient unless it is co-transcriptionally modified for effective and precise recognition (Fig 5D). Another layer of specificity in this regulation comes into play in the form of cellular host factors. In this study, we identified YTHDF2 as a key player of this regulation. YTHDF2 protein belongs to a family of YTH proteins which bind m^6^A modified RNAs. Interestingly, CCR4-NOT complex was found to be responsible for deadenylation and YTHDF2 can interact and thus destabilizes mRNA by accelerating deadenylation [19]. This suggests that m^6^A readers can interact with other RNA destabilizing factors and this study establishes ISG20 as another example of host factor that can selectively destabilize viral RNA by acting as YTHDF2 interacting partner. Using co-immunoprecipitation (co-IP) method and confocal microscopy, cellular YTHDF2 protein is shown to interact with ISG20 (Fig 2). Importantly, elimination of YTHDF2 neutralizes the action of ISG20 demonstrating that the presence of an m^6^A reader is essential for ISG20 to exert its effect. Despite the presence of ISG20 and a methylated ISG20 binding site, IFN-α does not have an effect, if YTHDF2 is depleted in HBV expressing cells (Fig 4A). In summary, we propose that ISG20 sensitivity and specificity are regulated by three major layers of control, in which 1) the presence of an appropriate binding site (such as GGACA within HBV ε loop), 2) appropriate epitranscriptomic modification (such as m^6^A herein) and 3) the presence of suitable host factors (such as YTHDF2) which coordinates the complex formation and facilitates the exonuclease action of ISG20 towards the transcripts of non-self-origin. While this scheme appears to be specific for HBV, it may have broader implications for other viral RNAs, in which additional factors may participate in IFN-mediated transcript decay.

The ε stem loop is present at 3´ termini of all the HBV transcripts while the 3.5 kb RNA species possesses this secondary structure at 5´ terminus as well. It is interesting to note here that the ISG20 is a 3´-5´ exonuclease and recognition of 3´ ε stem loop (present in all HBV transcripts) by ISG20 could possibly be a straightforward upstream event that can eventually initiate HBV RNA degradation via 3´-5´ exonuclease activity. However, our data clearly indicated that the 5´ ε stem loop also plays a similar role. It would be relevant to ask here, if ISG20 can also recognize 5´ ε stem loop and degrade HBV RNA in a similar fashion how binding of ISG20 at 5´ ε stem loop can then support 3´-5´ exonuclease degradation of HBV RNA, which requires the degradesome to be transferred to the 3´ end. The existence of Φ and ω elements within 3.5 kb species of HBV transcripts may partially settle this issue. The element Φ is situated 30 nt upstream of 3´ DR1 while the ω is present within the 3´ end of 3´ DR1 [35–39]. These two elements situated towards 3´ terminus of the transcripts, juxtapose 5´ and 3´ ends within close proximity [40]. With this proposed model, even if ISG20 recognizes and binds with 5´ ε stem loop, it will still be in a close proximity of 3´ terminal to execute final 3´-5´ degradation (Fig 5E). Our proposed model therefore suggests that the ISG20 binding with any ε stem loop (5´ or 3´) is basically an initial recognition step, which is critically regulated by m^6^A modification and YTHDF2 protein. Therefore it is clear that once ISG20- ε stem loop interaction is established, regardless of which terminus, the 3´ end of the transcripts will always be accessible to the degradesome. However, we cannot rule out the possibility that factors other than ISG20 and YTHDF2, are also required for the formation of complete degradesome. In summary, our study provides molecular insights into IFN-α induced RNA degradation scheme, thus opening up new possible therapeutic avenues exploring epitranscriptomic modification of HBV transcripts as possible targets to prevent cccDNA formation and associated viral persistence in HBV patients.

## Materials and methods

### Cell culture and transfection

HepG2 cells were maintained in Dulbecco’s modified Eagle’s medium (DMEM) supplemented with 10% Fetal Bovine Serum (FBS). Plasmids were transfected into HepG2 cells using Mirus TransIT-LT1 reagent (Mirus, USA) according to the manufacturer’s protocol. For siRNA mediated knockdown of METTL3, METTL14, and YTHDF2, siGENOME Human METTL3 (56339) siRNA-SMARTpool, siGENOME Human METTL14 (57721) siRNA-SMARTpool, and siGENOME Human YTHDF2 (51441) siRNA-SMARTpool (Dharmacon, USA) were used; the ON-TARGET plus non-targeting pool (D-001810-10-05) was used as the scrambled control. Lipofectamine RNAiMAX reagent was used for siRNA transfection (Thermo Fisher Scientific, USA) according to the manufacturer’s protocol.

### Plasmids and antibodies

HBV 1.3mer plasmid was a kind gift from Dr. Wang-Shik Ryu and obtained from the Addgene (65459). Anti-METTL3 antibody was purchased from Proteintech (USA) and anti-METTL14 from Sigma (USA), anti-YTHDF2 from Abcam (USA), anti-HA and anti-FLAG antibodies were from Cell Signaling Technologies (USA), anti-GAPDH and anti-HBsAg were from Santa Cruz Biotechnology (USA), anti-Rabbit IgG HRP and anti-mouse IgG HRP antibodies were from Promega (USA) and anti-m^6^A antibody was obtained from Synaptic Systems (Germany).

### Co-Immunoprecipitation (co-IP) assay

Cell pellets were lysed with 1X Lysis buffer (20 mM Tris HCl;pH 8.0, 137 mm NaCl, 1% NP-40 and 2 mM EDTA), kept on ice for 30 min, centrifuged at 13,000 rpm for 15 min and the supernatant was taken for Bradford assay. 500μg lysates were mixed with protein A/G beads and rotated for 1 h and then centrifuged at 2,000 rpm for 2–3 min at 4°C. To the clarified supernatant, 1 μg of antibody was added and kept at 4°C for overnight in a rotator. To this 30 μl of A/G beads were added and the mixing continued for 2–3 h. The mixture was centrifuged at 2,000 rpm for 2–3 min at 4°C and the pellet was washed thrice gently with 1X lysis buffer. The washed pellet was resuspended in 50 μl of SDS loading dye and boiled for 5 min. After centrifugation at 13,000 rpm for 5 min and the upper layer was used to load in SDS-PAGE gel to perform western blot assay.

### Western blot assays

Cell pellets were washed twice with ice-cold PBS, lysed with 1X RIPA buffer (1M Tris HCl;pH 7.4, 2.5 M NaCl, 10% NP-40, 10% DOC, 1% Protease inhibitor cocktail, 10% SDS), kept in ice for 30 min and then centrifuged at 13,000 rpm for 15 min. After protein estimation, SDS loading dye was added to the samples and boiled at 95°C for 10 min. Clarified lysates were resolved by SDS-PAGE Gel and transferred to nitrocellulose membranes (BioRad, USA). The membrane was blocked with 5% BSA or 5% non-fat milk for 1 h, followed by overnight incubation with primary antibodies (1:1000) diluted in 2% BSA or 5% non-fat milk. After washing, the membranes were incubated with HRP conjugated secondary antibodies (1:5000) for 1 h. The signals were detected using a chemiluminescence substrate (Thermo Scientific, USA).

### Immunofluorescence

HepG2 cells, grown on coverslips were transfected with indicated plasmids followed by immunofluorescence assay, as described previously [41]. Under 60x or 100x oil objectives, cells were visualized using an Olympus FluoView 1000 confocal microscope. Images were quantified with Image J, Adobe and MBF Image J software.

### Quantitative PCR

RNA was isolated using RNeasy mini kit (Qiagen, USA). m^6^A modified RNA was immunoprecipitated according to the protocol described previously [42]. iScript Reverse Transcription Supermix for RT-qPCR (BioRad, USA) was used to prepare cDNA and quantitative PCR was done with SsoAdvanced Universal SYBR Green Supermix (BioRad, USA) using the following primers: HBV-specific primer forward, 5´-CTCAATCTCGGGAATCTCAATGT -3´, HBV-specific primer reverse, 5´- TGGATAAAACCTGGCAGGCATAAT -3´, GAPDH forward, 5´-TGCACCACCAACTGCTTAGC-3´, GAPDH reverse, 5´-GGCATGGACTGTGGTCATGAG-3´. The RT-qPCR program was 95°C for 3 min followed by 40 cycles at 95°C for 10 sec, 58°C for 30 sec, and fold changes in gene expression were calculated by the ΔΔCT method.

## Supporting information

S1 Fig Schematics indicate the location of the A1907C mutations of m^6^A sites in HBV RNAs. (TIF)

S2 Fig Both endogenous ISG20 (IFN-α induced) and mutated ISG20 (HA-ISG20^D94G^) separately forms a complex with YTHDF2 but only endogenous ISG20/YTHDF2 complex degrades m^6^A modified HBV RNA. (TIF)
