# Correction: Interferon-stimulated gene 20 (ISG20) selectively degrades N6-methyladenosine modified Hepatitis B Virus transcripts

**DOI:** 10.1371/journal.ppat.1014336

**Published:** 2026-06-16

**Authors:** Hasan Imam, Geon-Woo Kim, Saiful Anam Mir, Mohsin Khan, Aleem Siddiqui

Contrary to the Data Availability statement in [1], the raw quantitative data underlying Figs 1, 3-4, and S1-2 were not provided as Supporting Information. The authors have provided the raw quantitative data, and the western blots underlying Figs 1-4 and S2, as S1 Data. With this correction, all relevant data are now provided.

## Supporting information

S1 Data Underlying data in support of [1]. (ZIP)
