# Supplementary figures and images for: Interferon-stimulated gene 20 (ISG20) selectively degrades N6-methyladenosine modified Hepatitis B Virus transcripts
Source: PLoS Pathog. 2020 Feb 14;16(2):e1008338. doi: 10.1371/journal.ppat.1008338 (PMC7046284; doi:10.1371/journal.ppat.1008338)

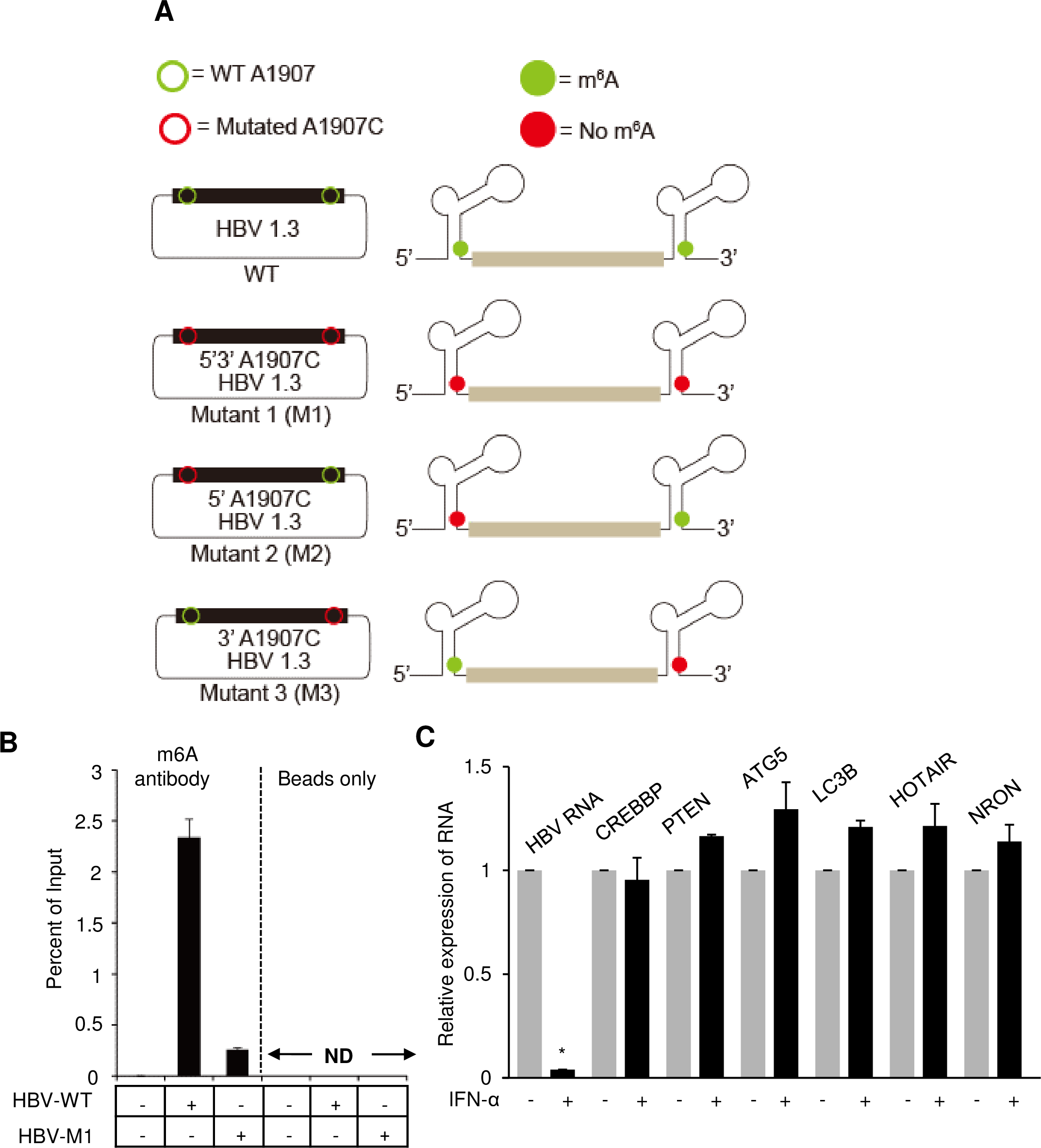

Supplement: S1 Fig — (TIF) [file ppat.1008338.s001.tif]

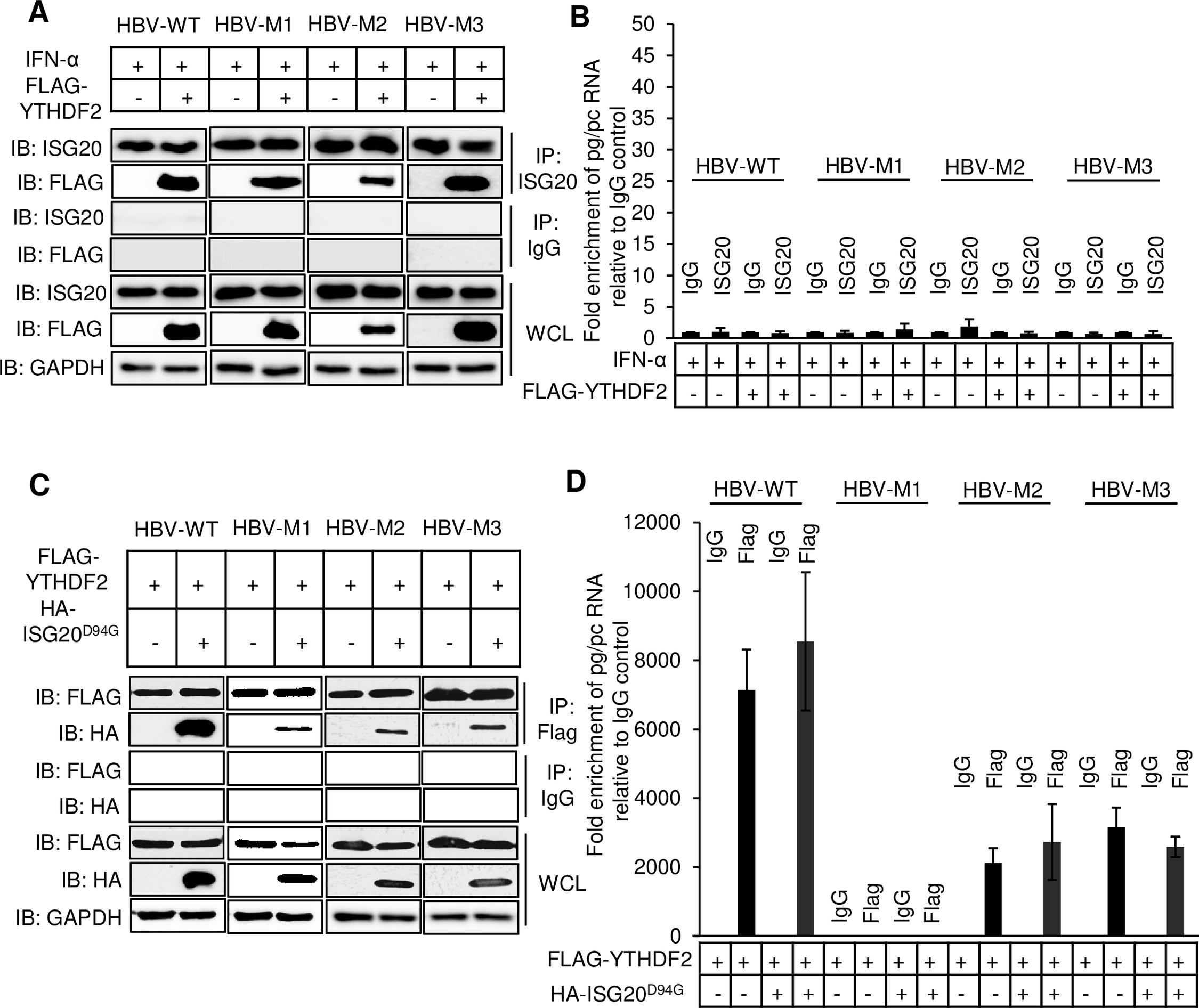

Supplement: S2 Fig — (TIF) [file ppat.1008338.s002.tif]
