# Supplementary material for: Correction: Interferon-stimulated gene 20 (ISG20) selectively degrades N6-methyladenosine modified Hepatitis B Virus transcripts
Source: PLoS Pathog. 2026 Jun 16;22(6):e1014336. doi: 10.1371/journal.ppat.1014336 (PMC13271447; doi:10.1371/journal.ppat.1014336)

Fig 1A

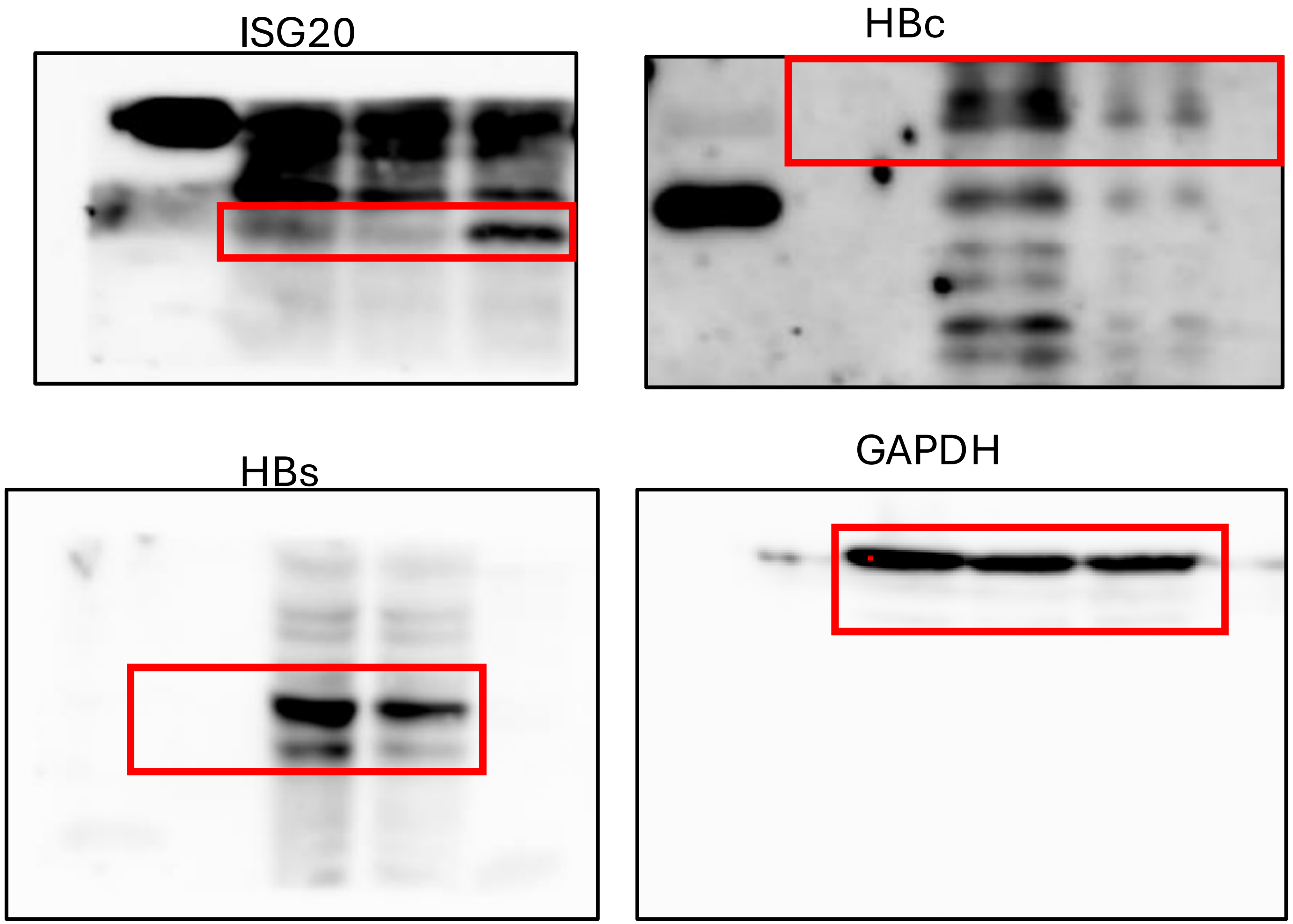

Fig 1B

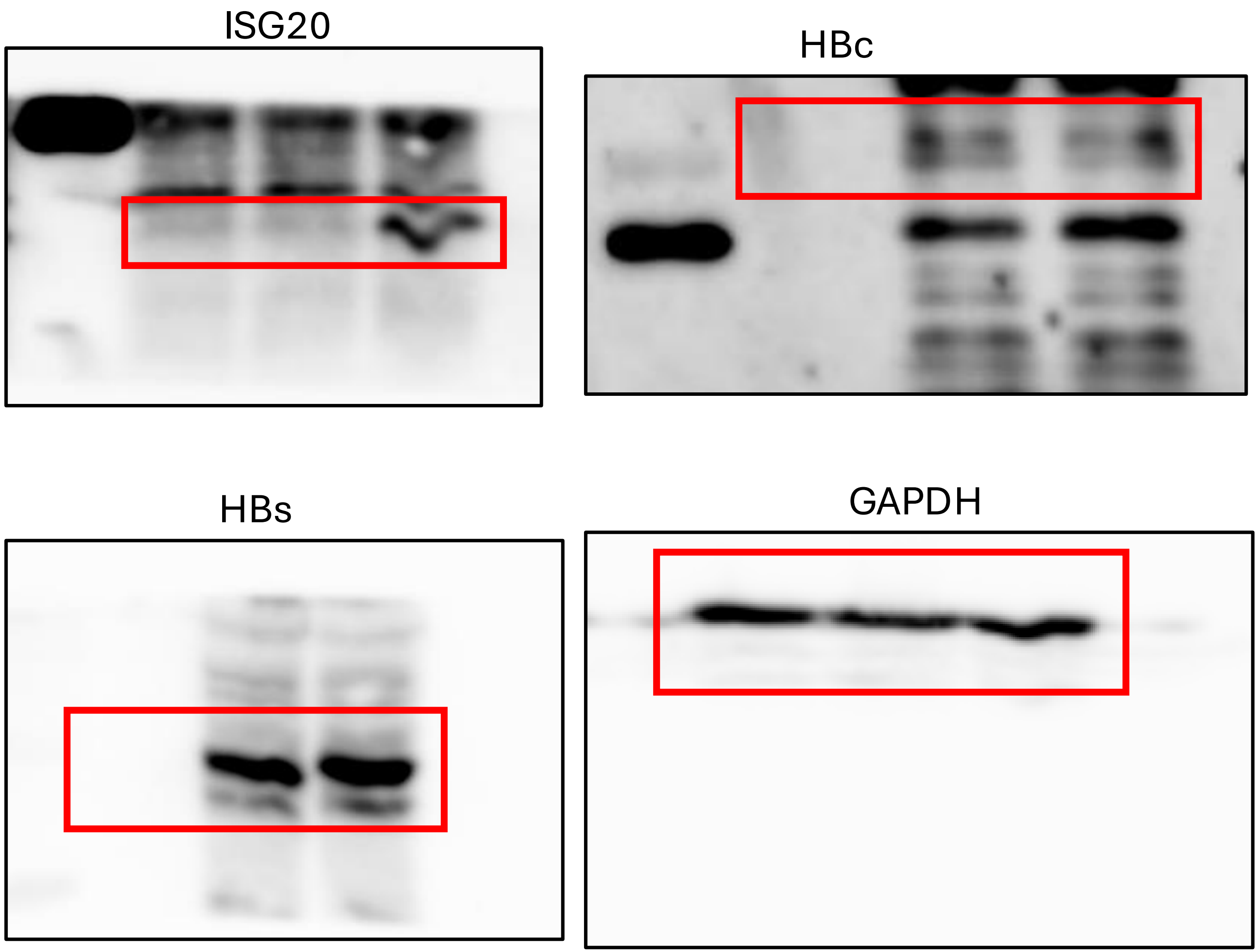

Fig 1C

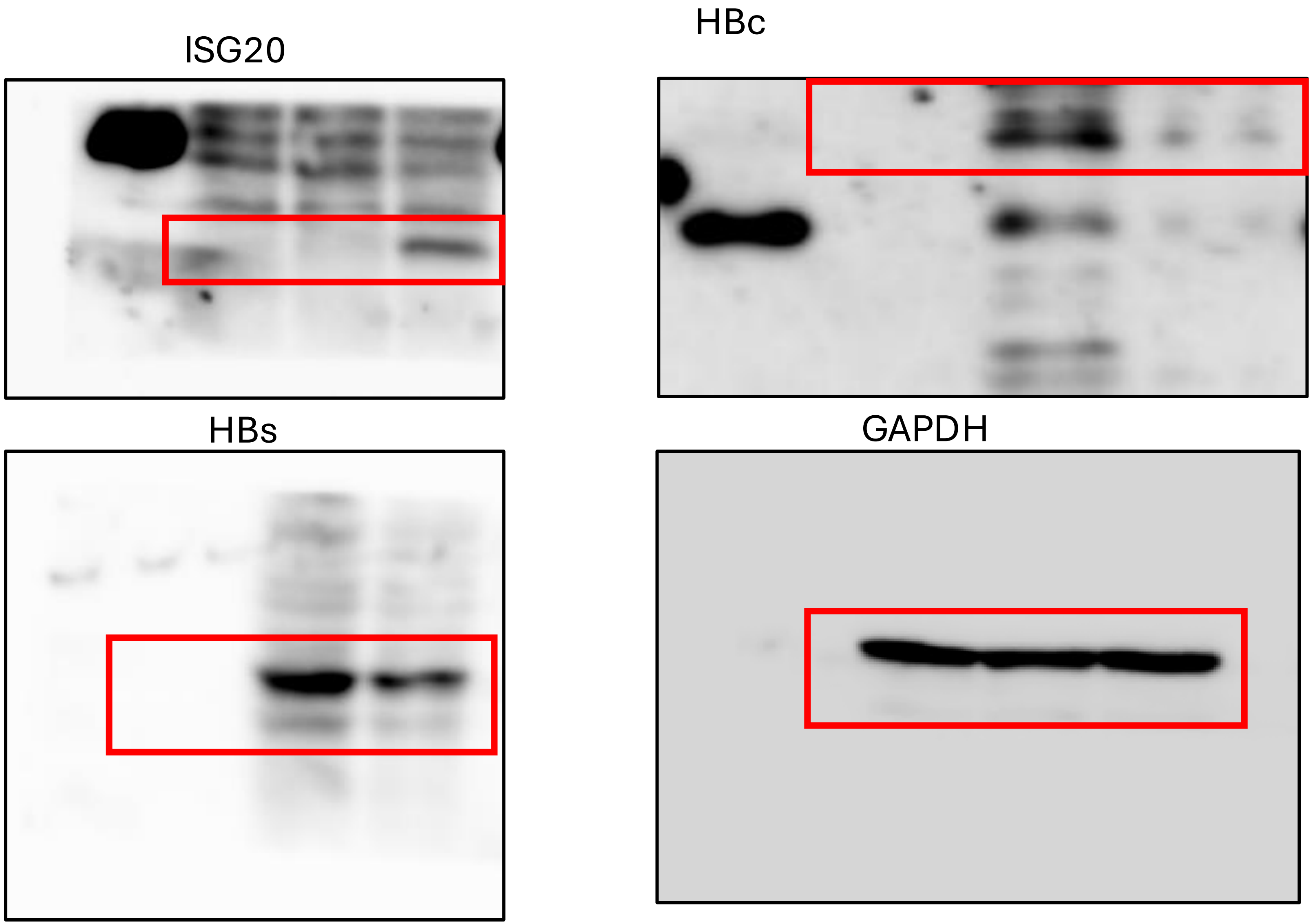

Fig 1D

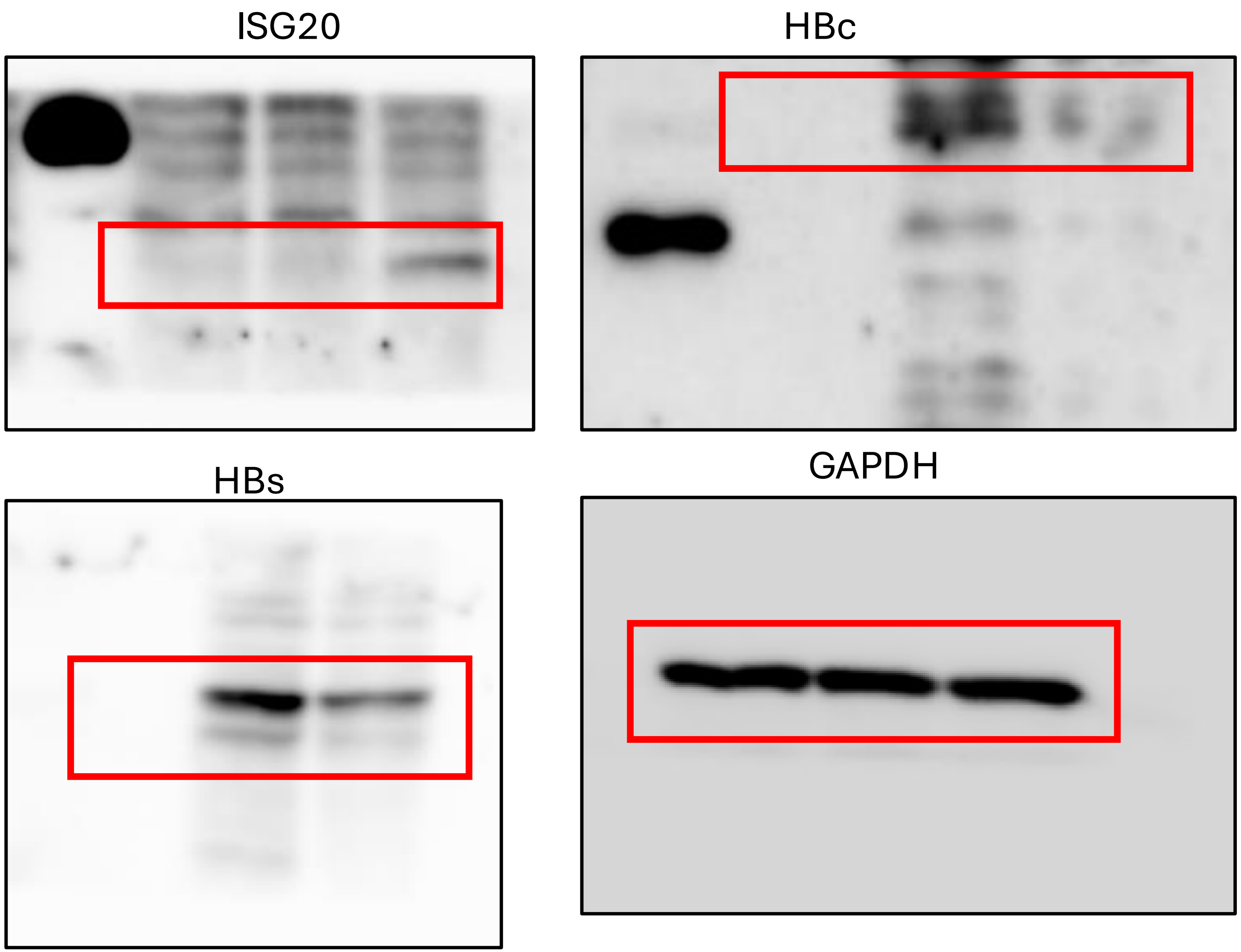

Fig 1E

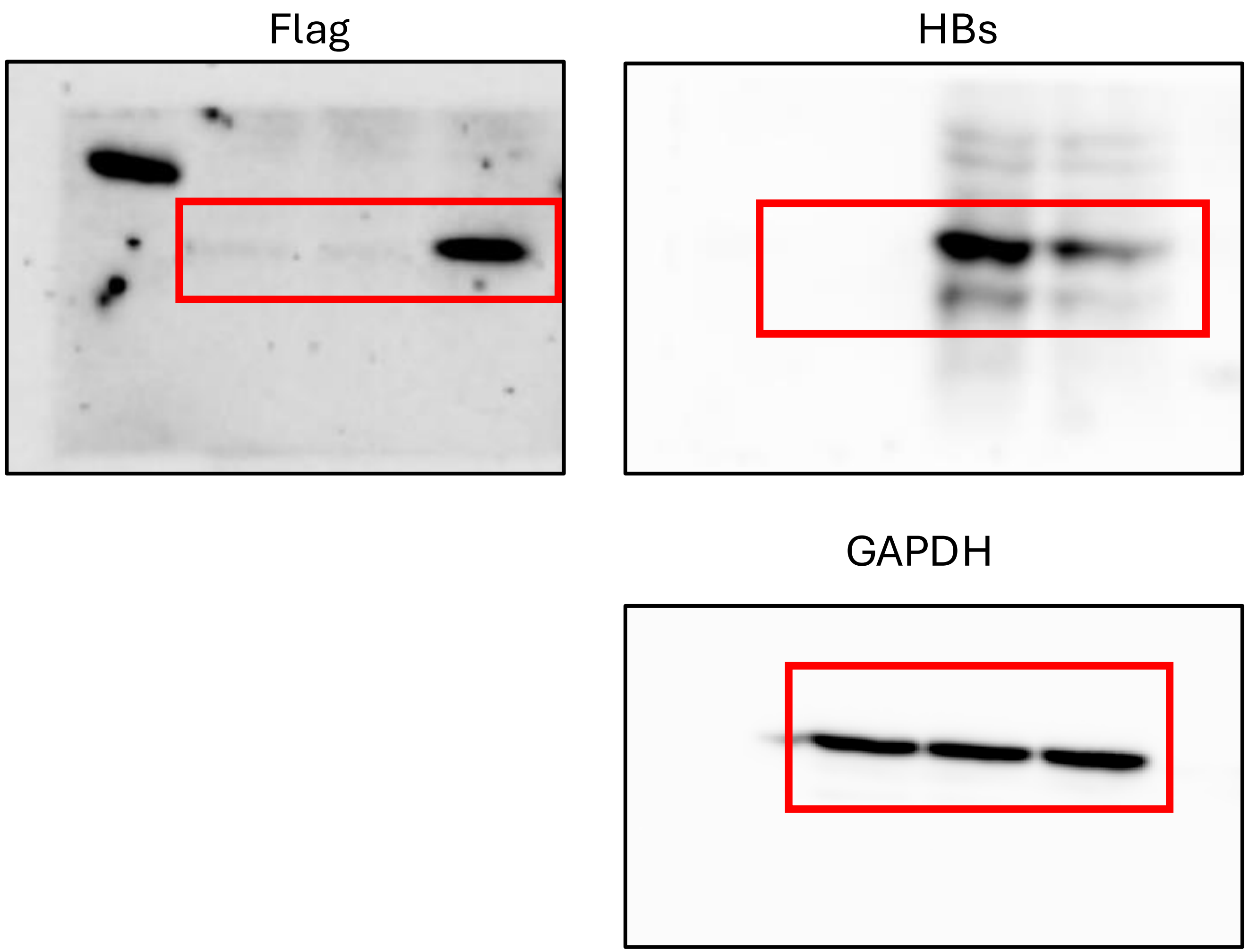

Fig 1F

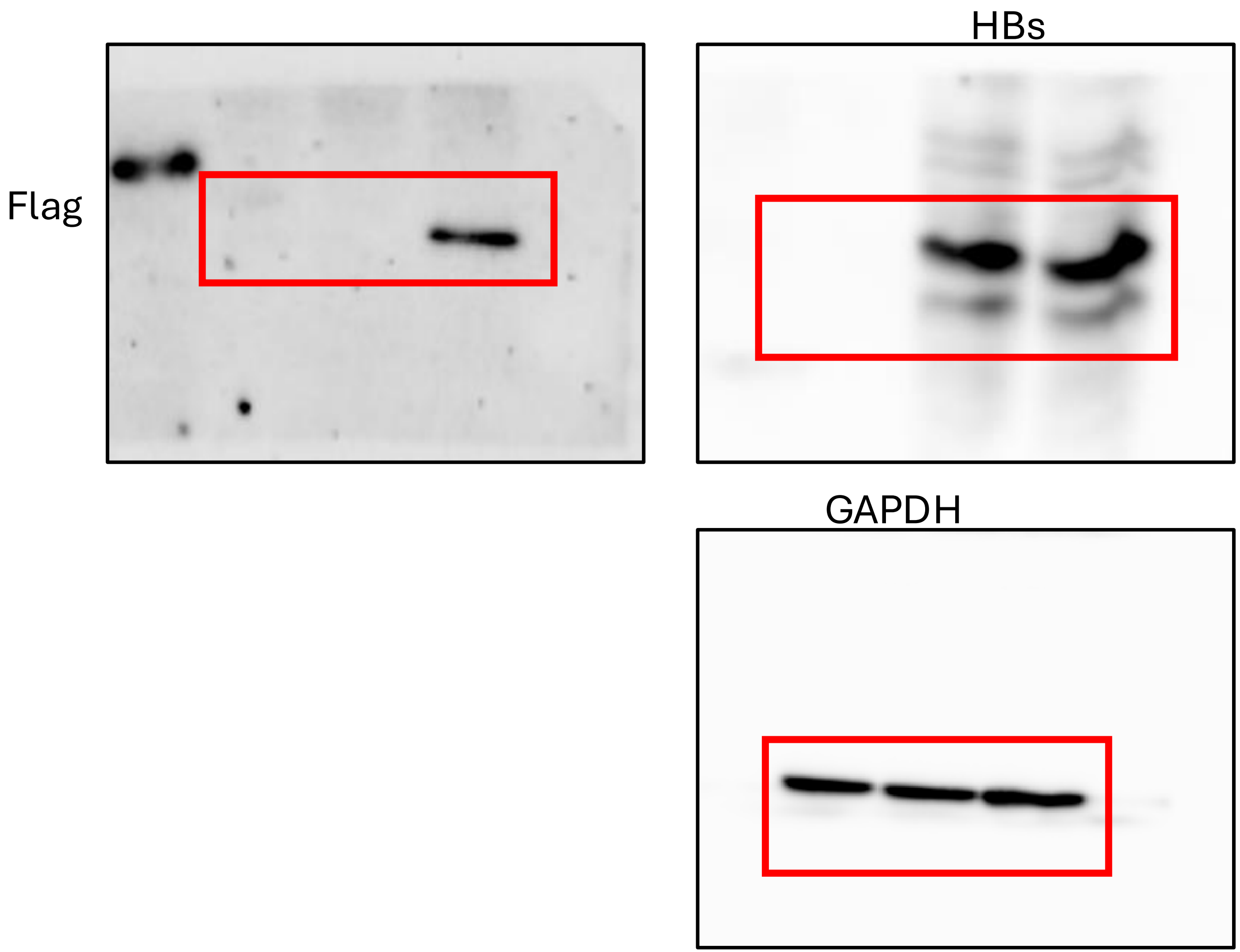

Fig 1G

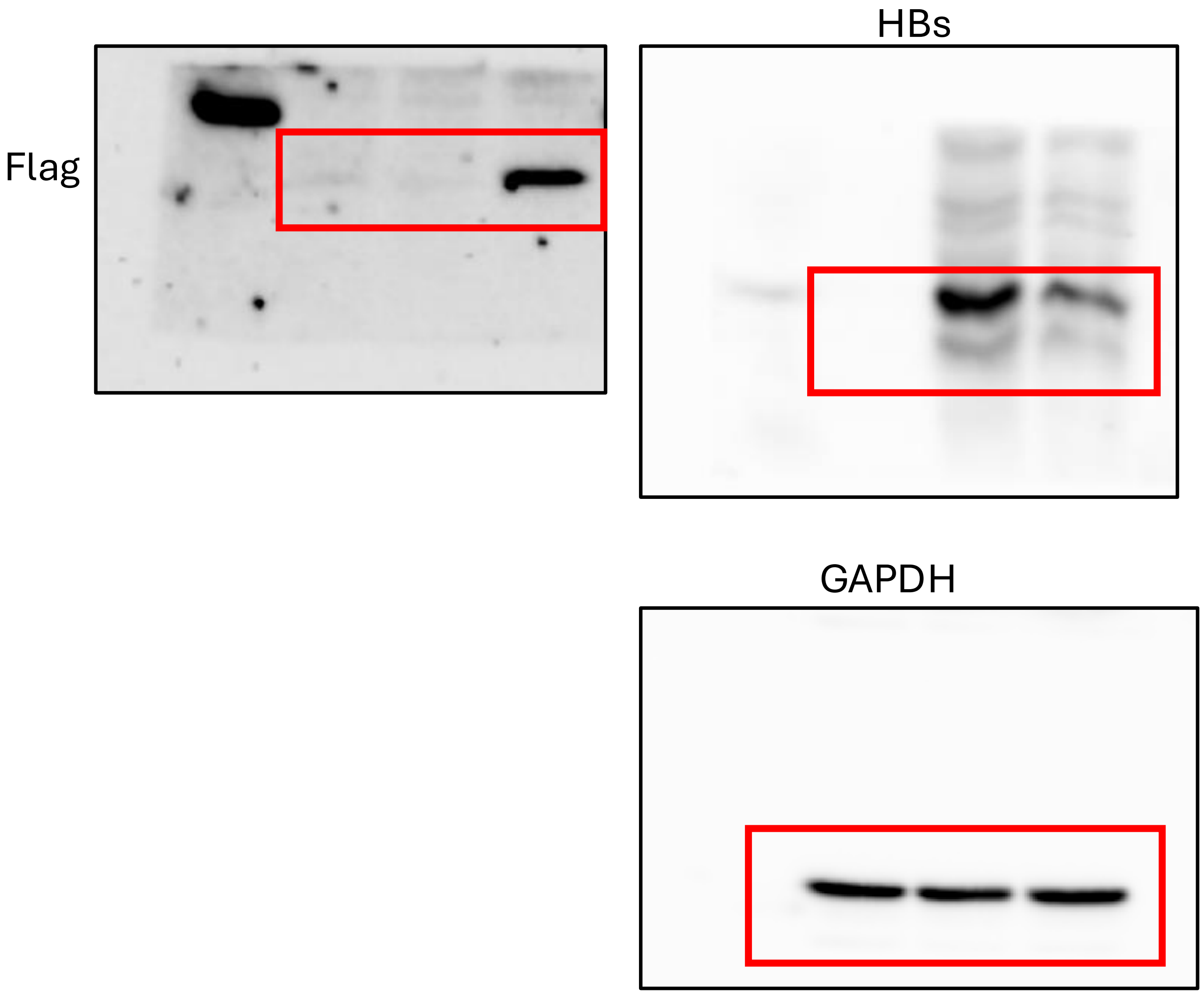

Fig 1H

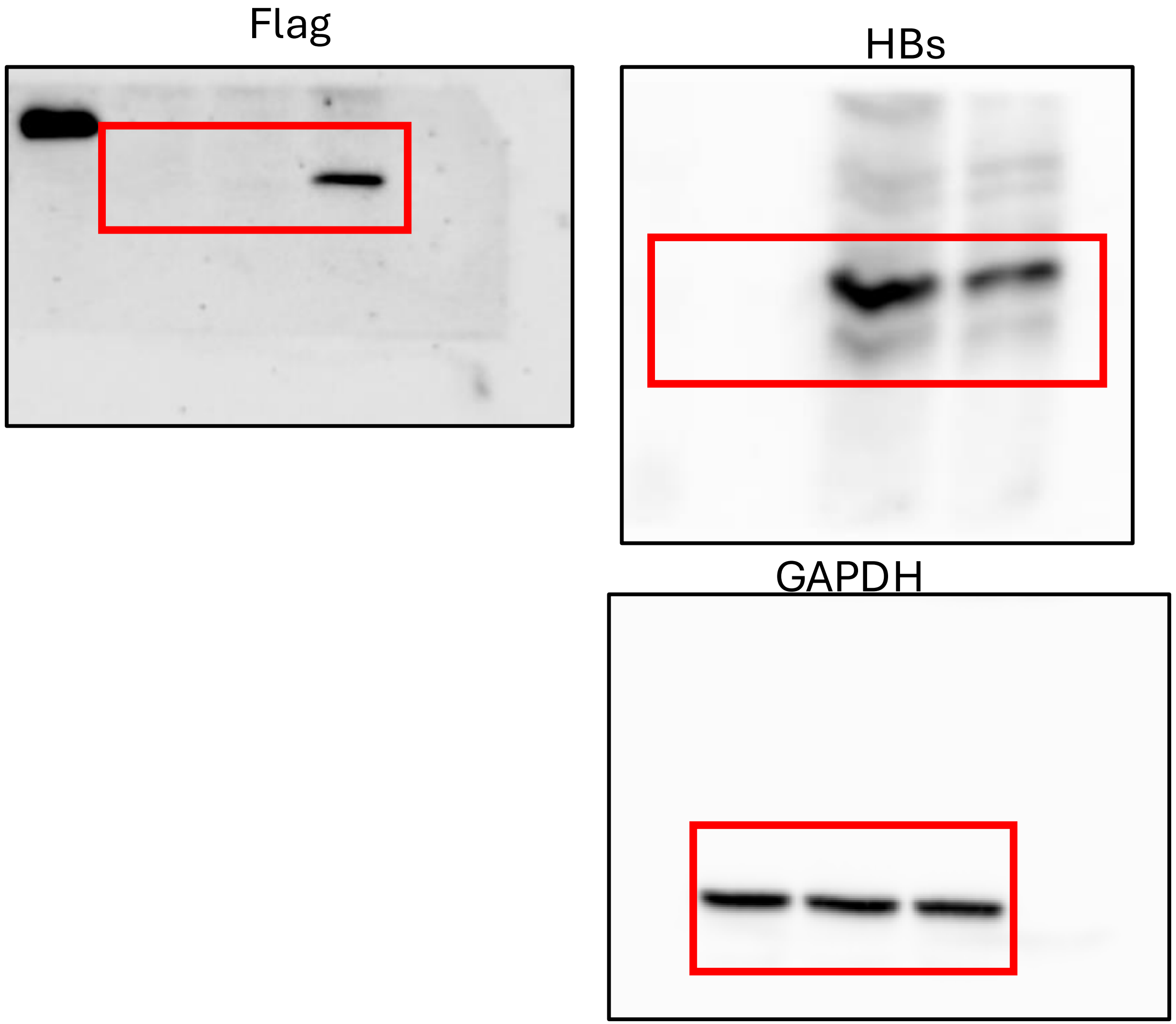

Fig 1I

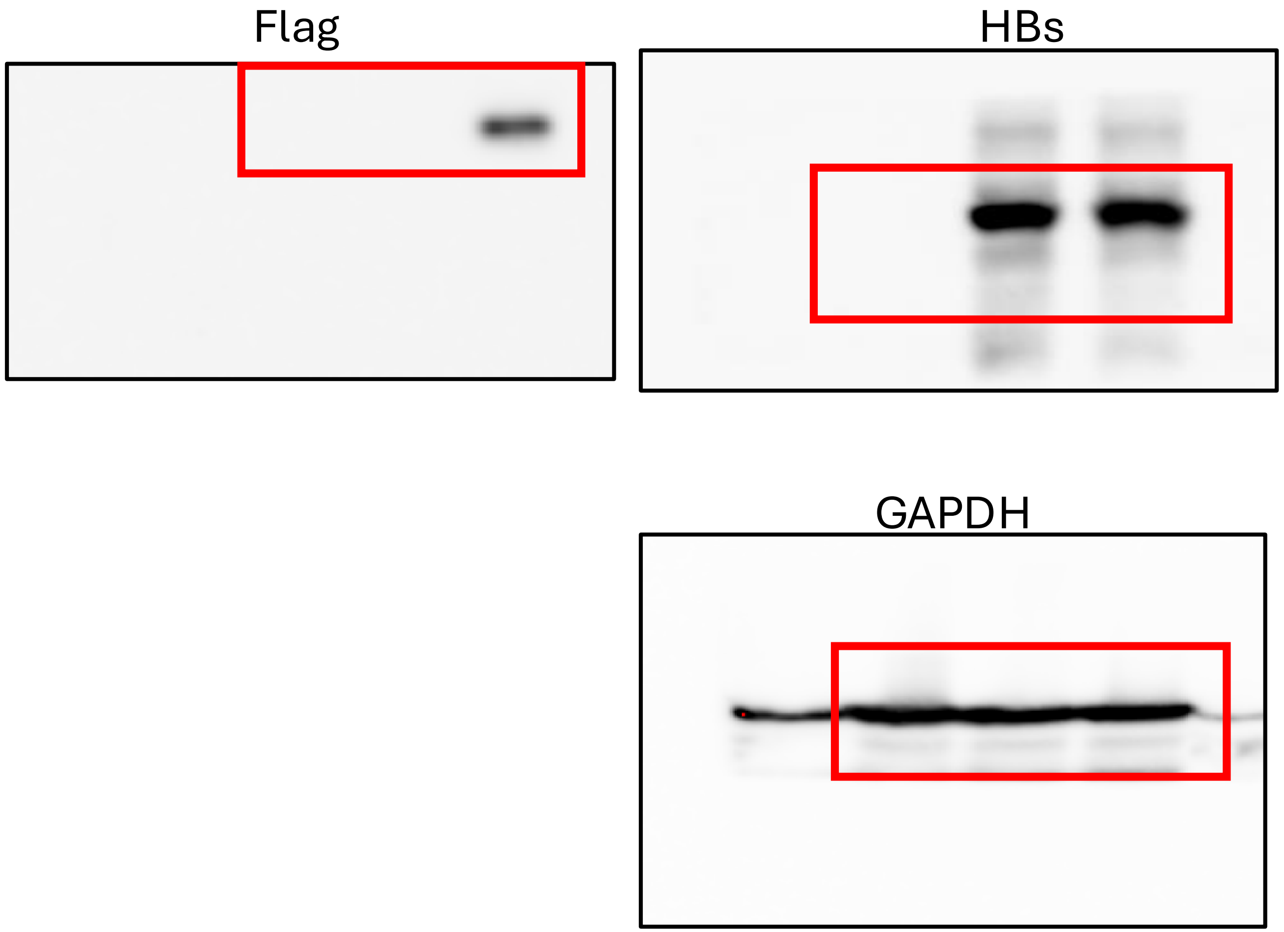

Fig 1J

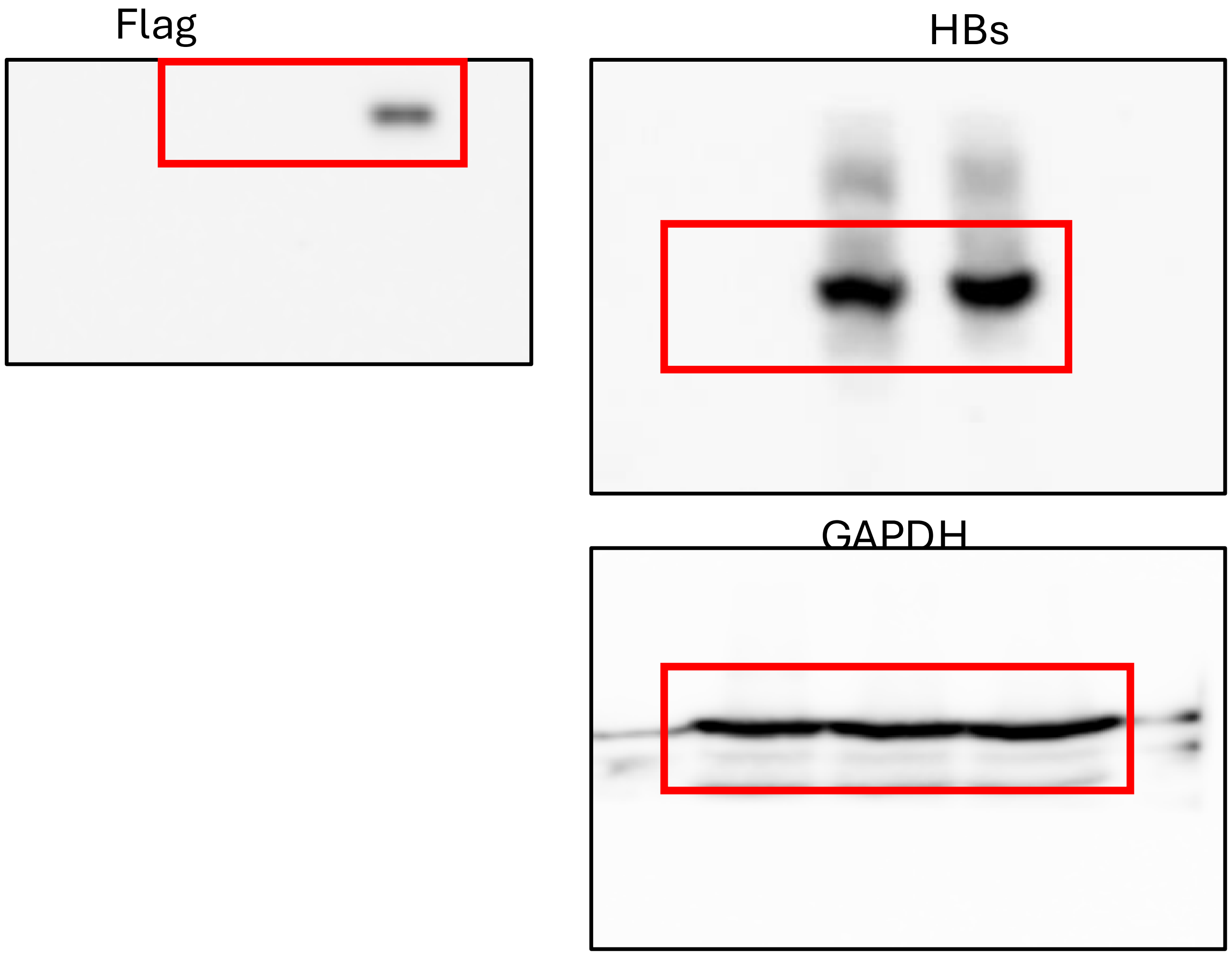

Fig 1K

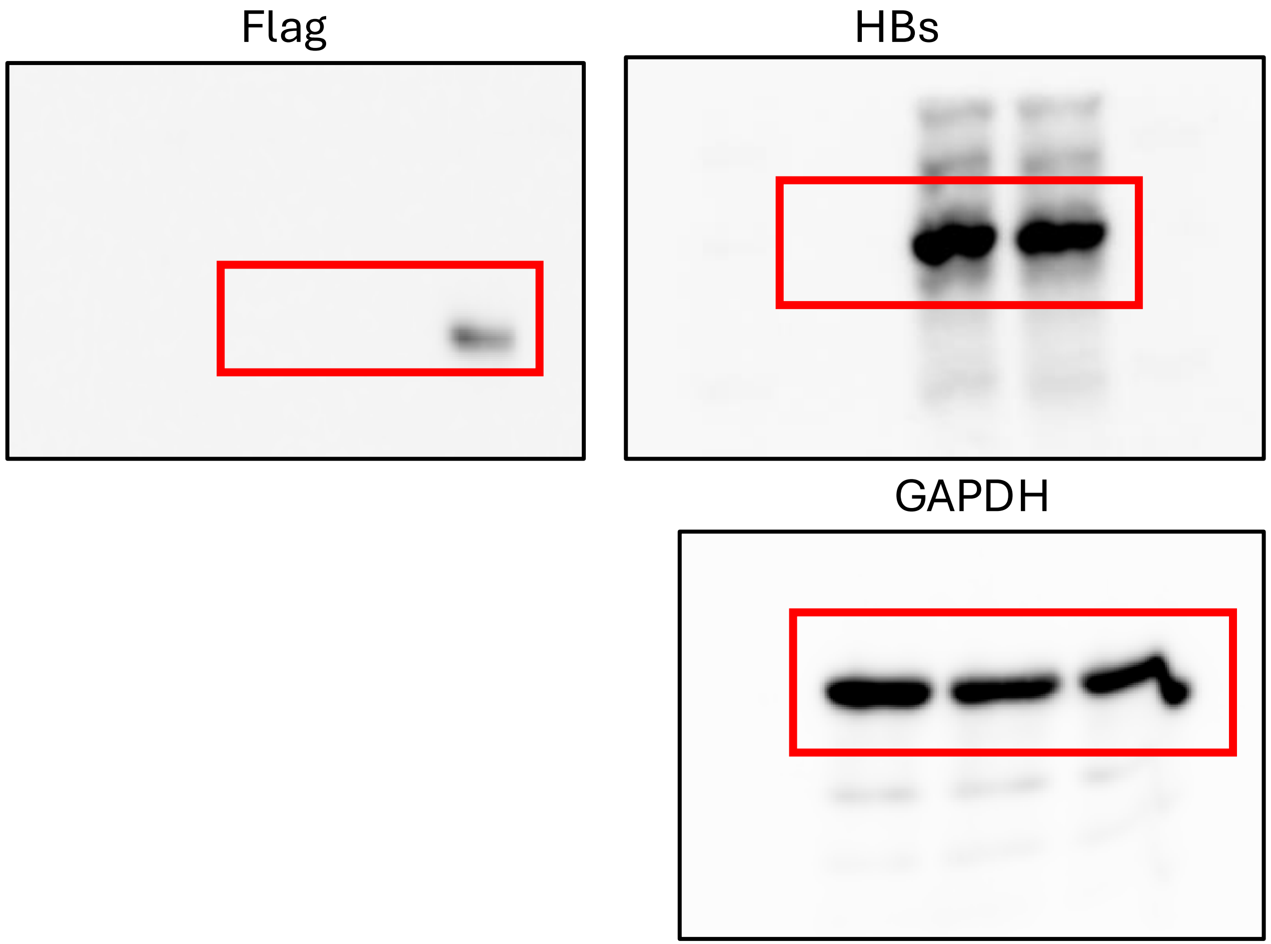

Fig 1L

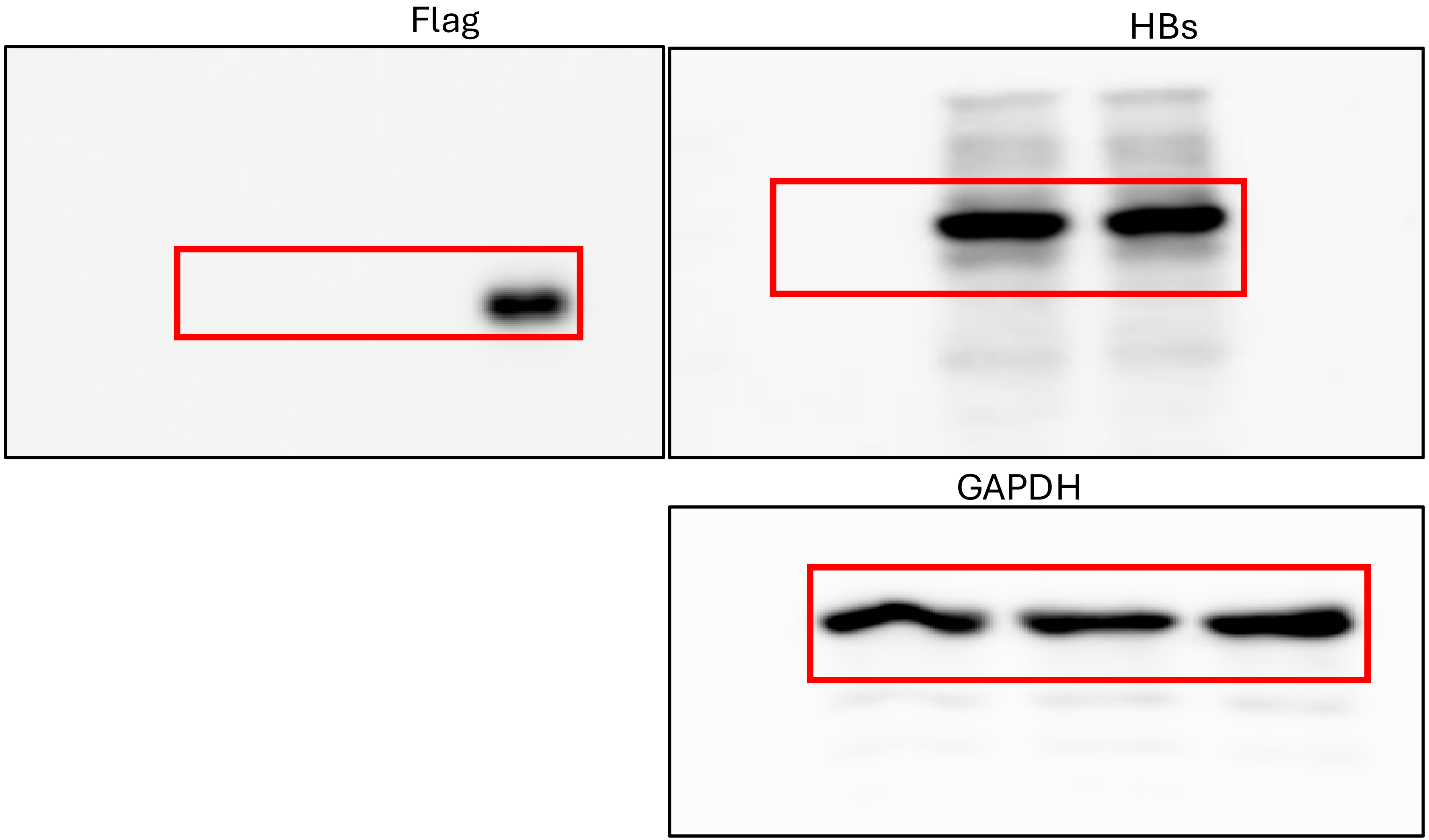

Fig 2A

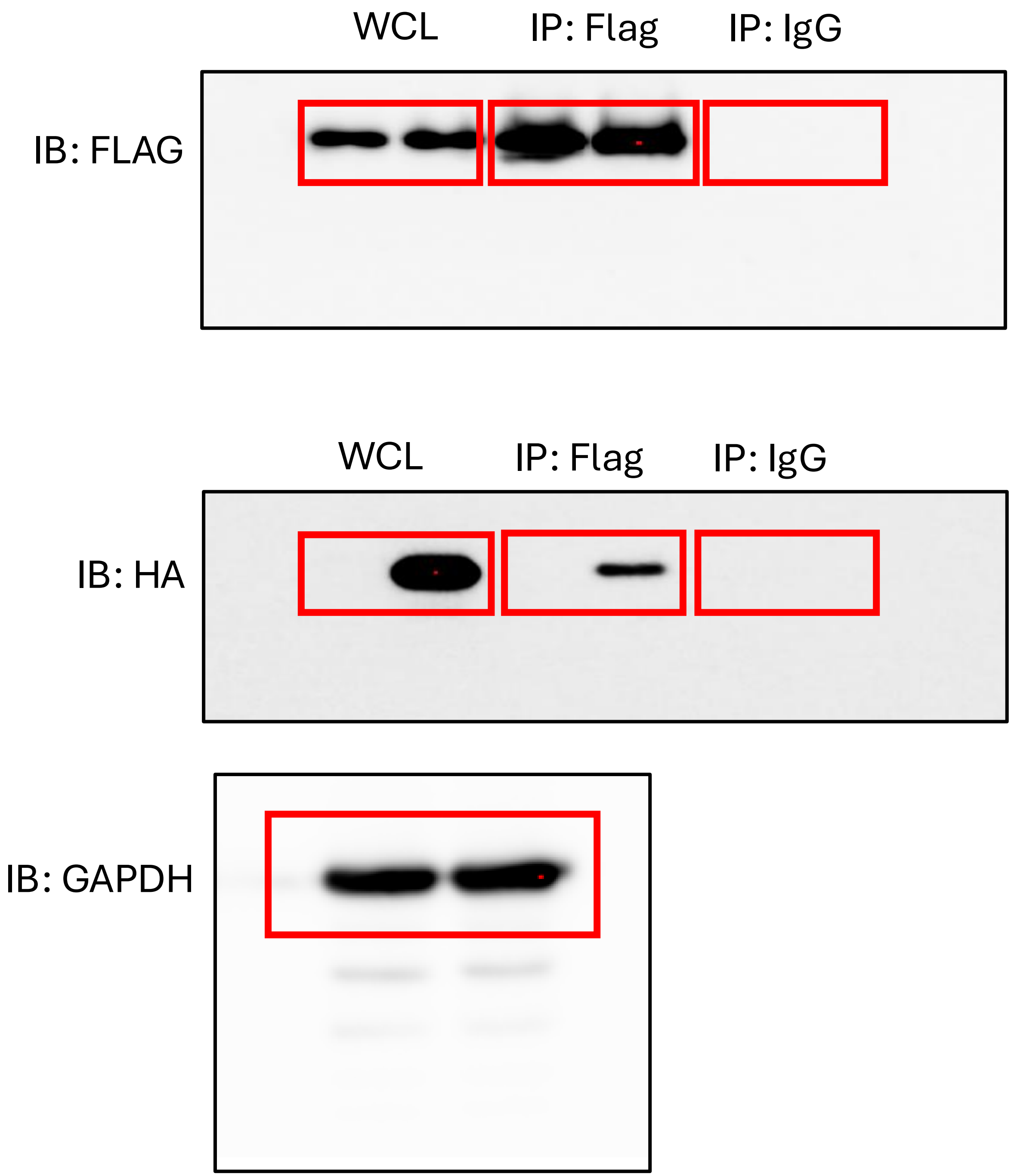

Fig 2B

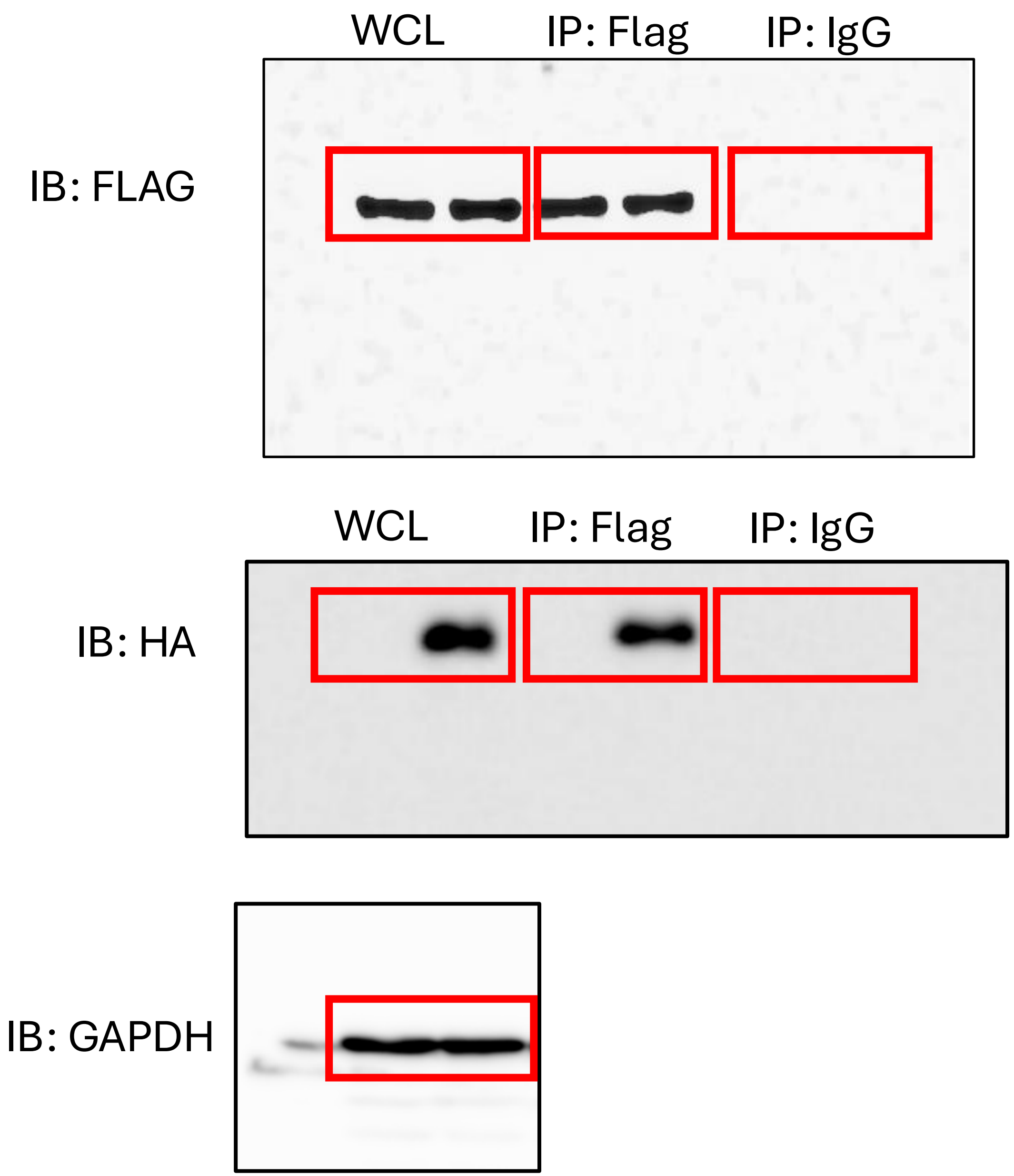

Fig 2C

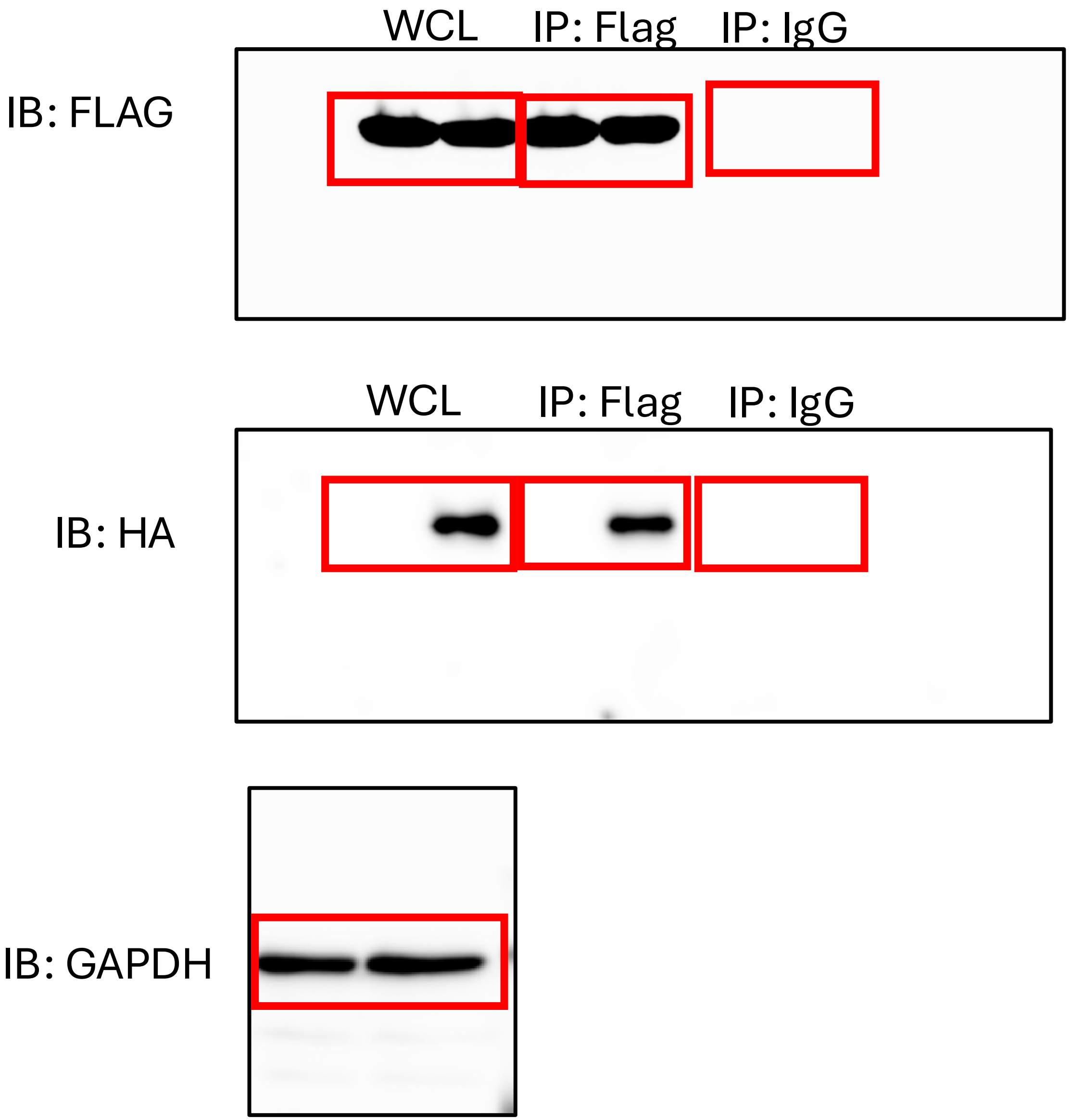

Fig 2D Upper Panel Left Half

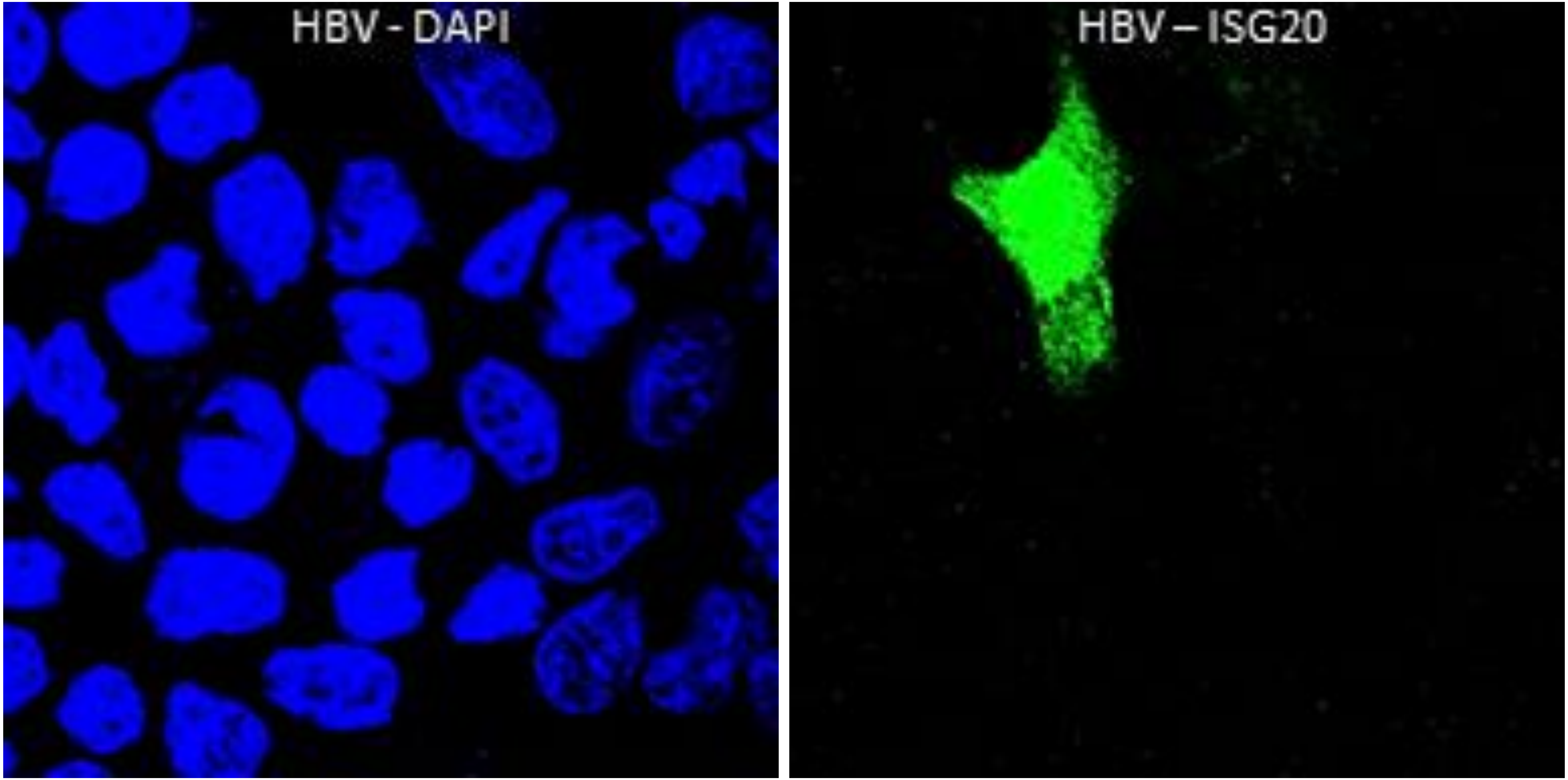

Fig 2D Upper Panel Right Half

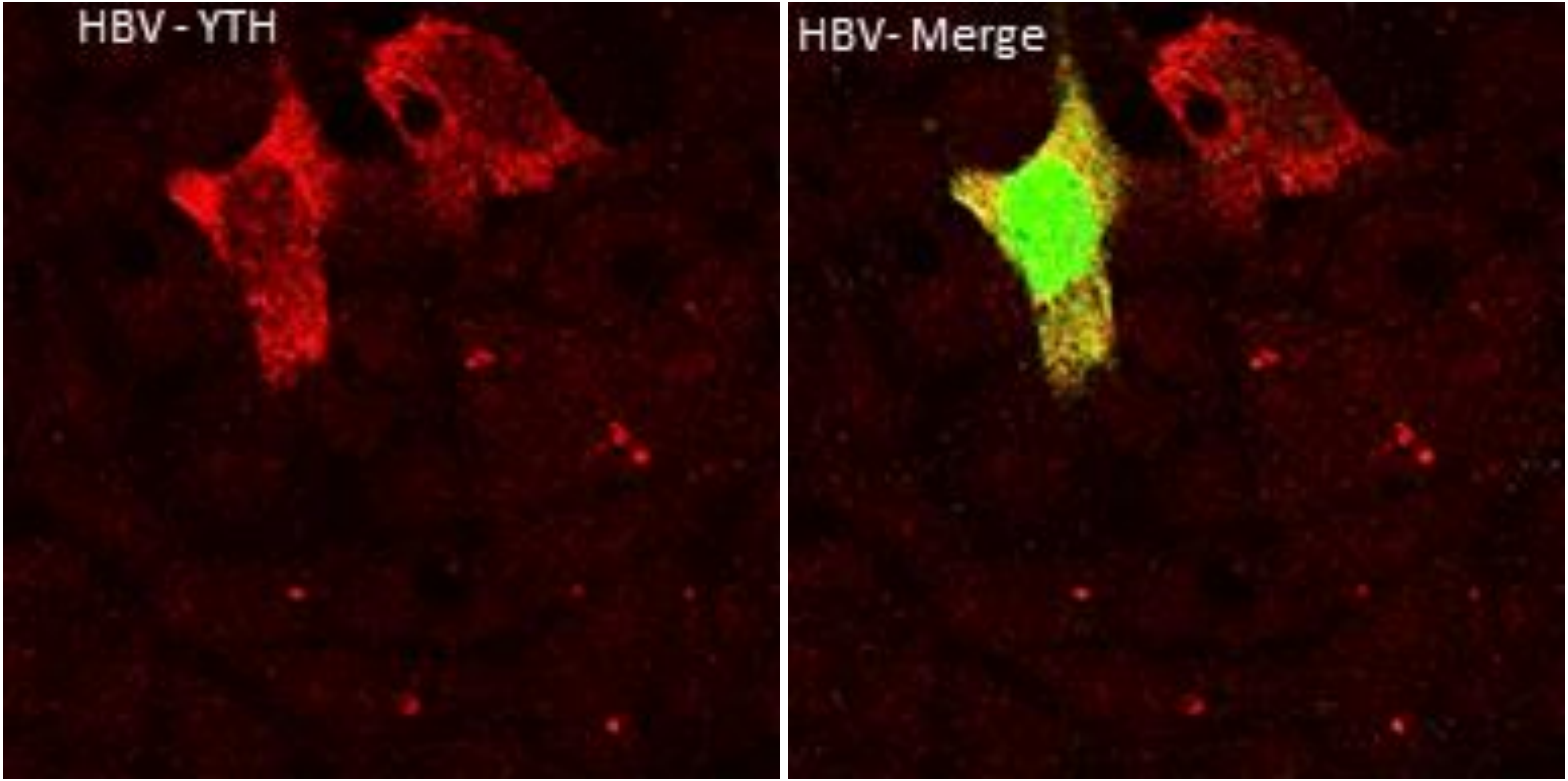

Fig 2D Lower Panel Left Half

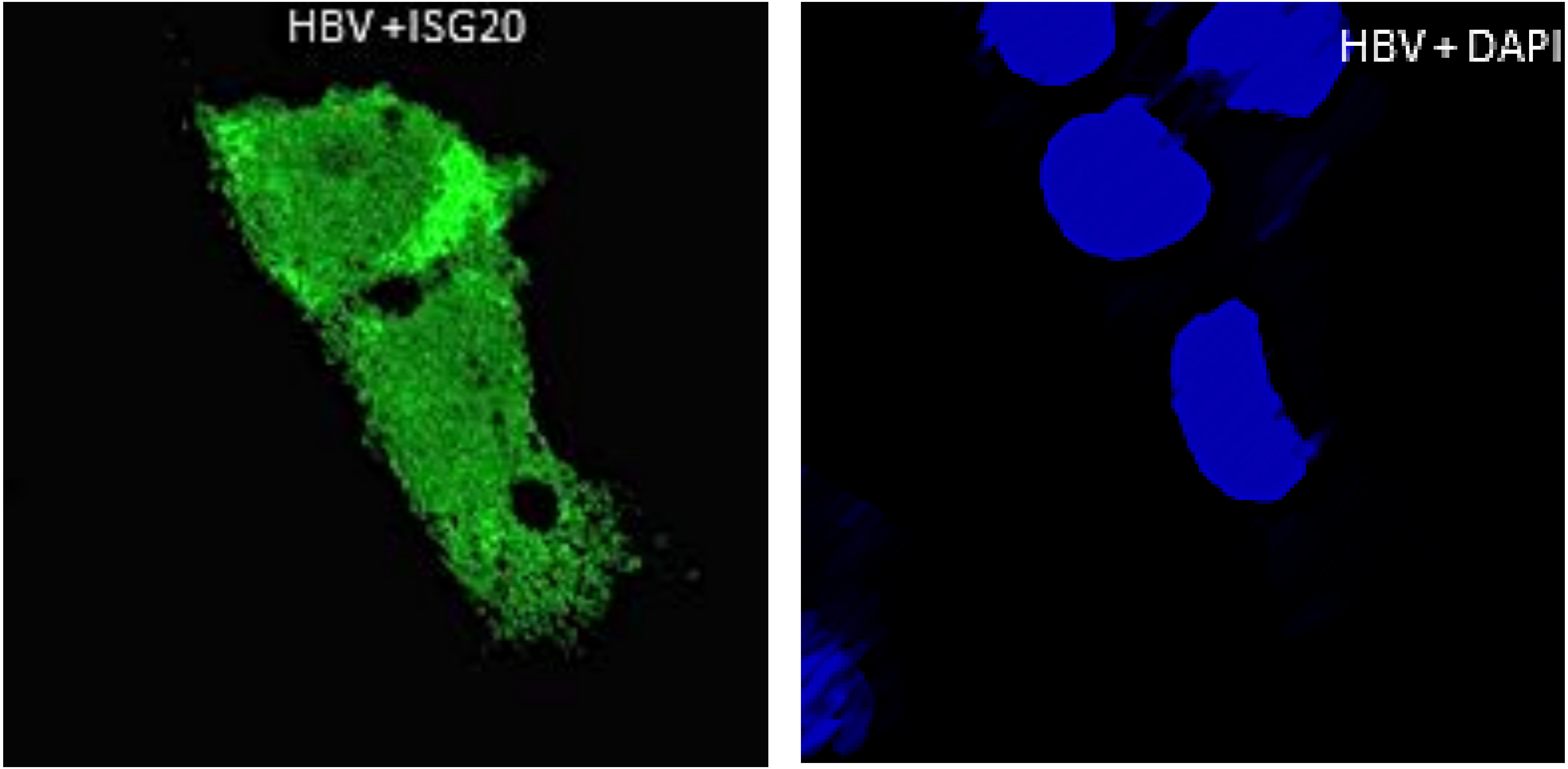

Fig 2D Lower Panel Rright Half

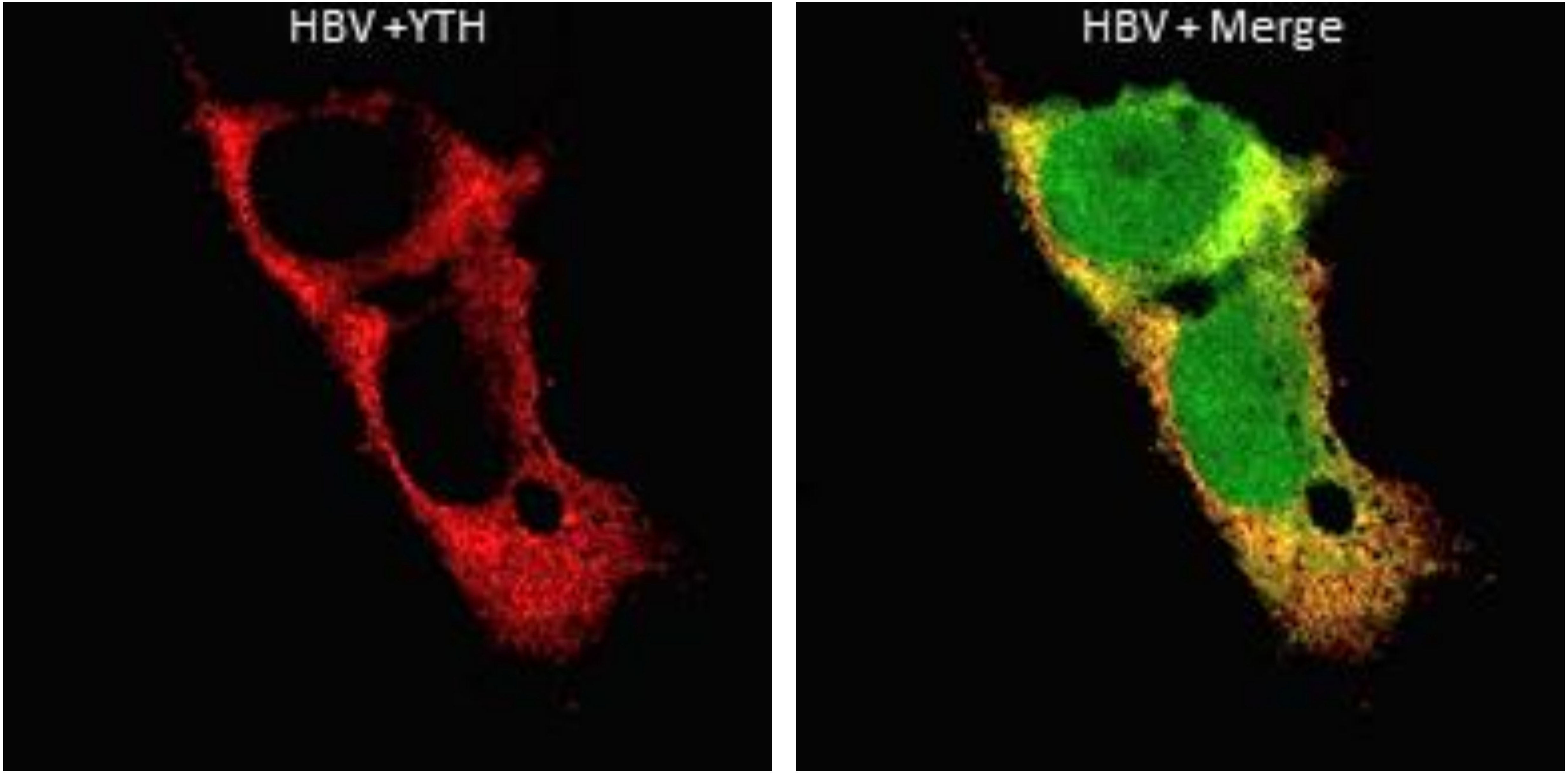

Fig 3 (1st Panel: HBV-WT)

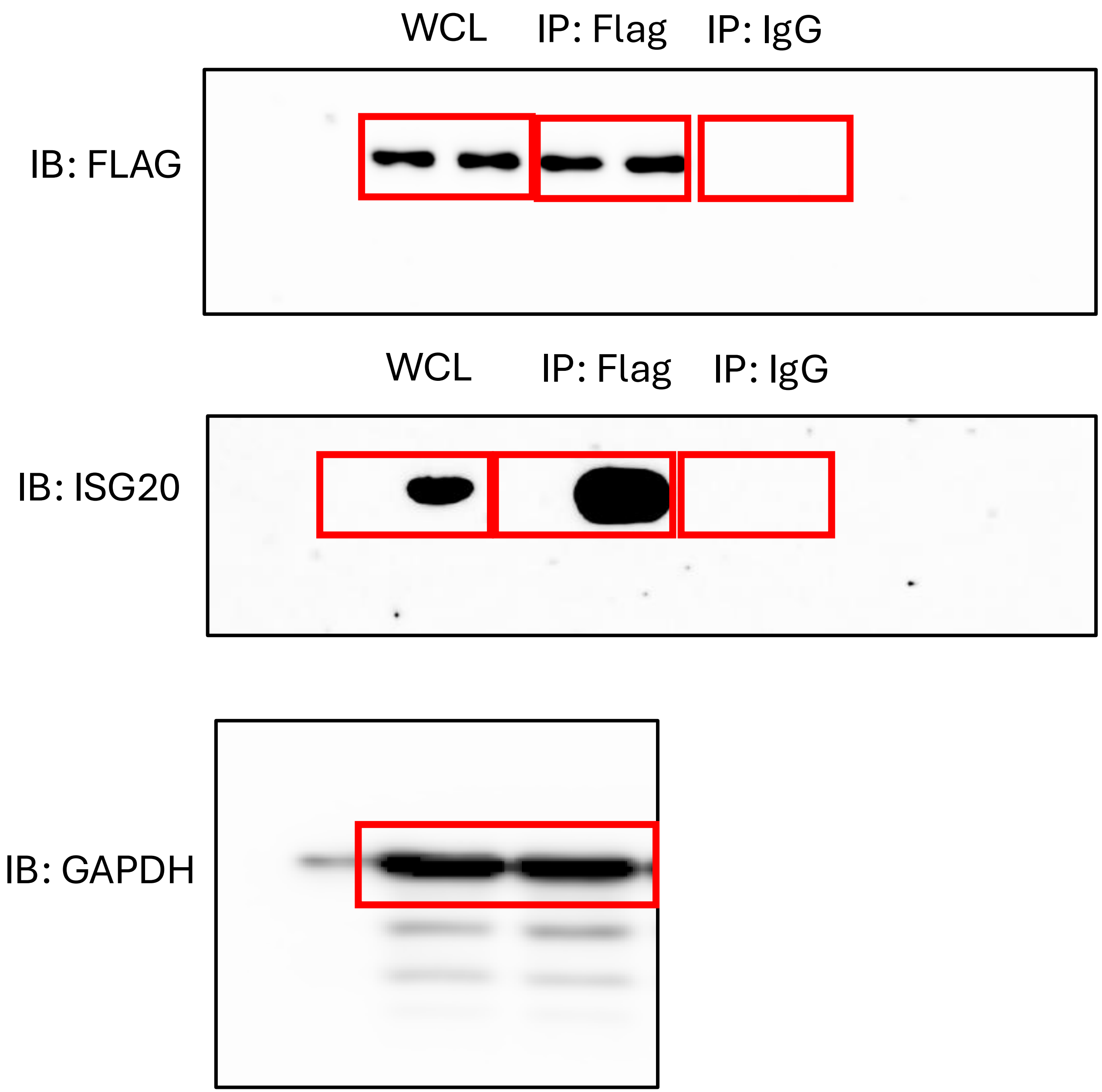

Fig 3 (2nd Panel: HBV-M1)

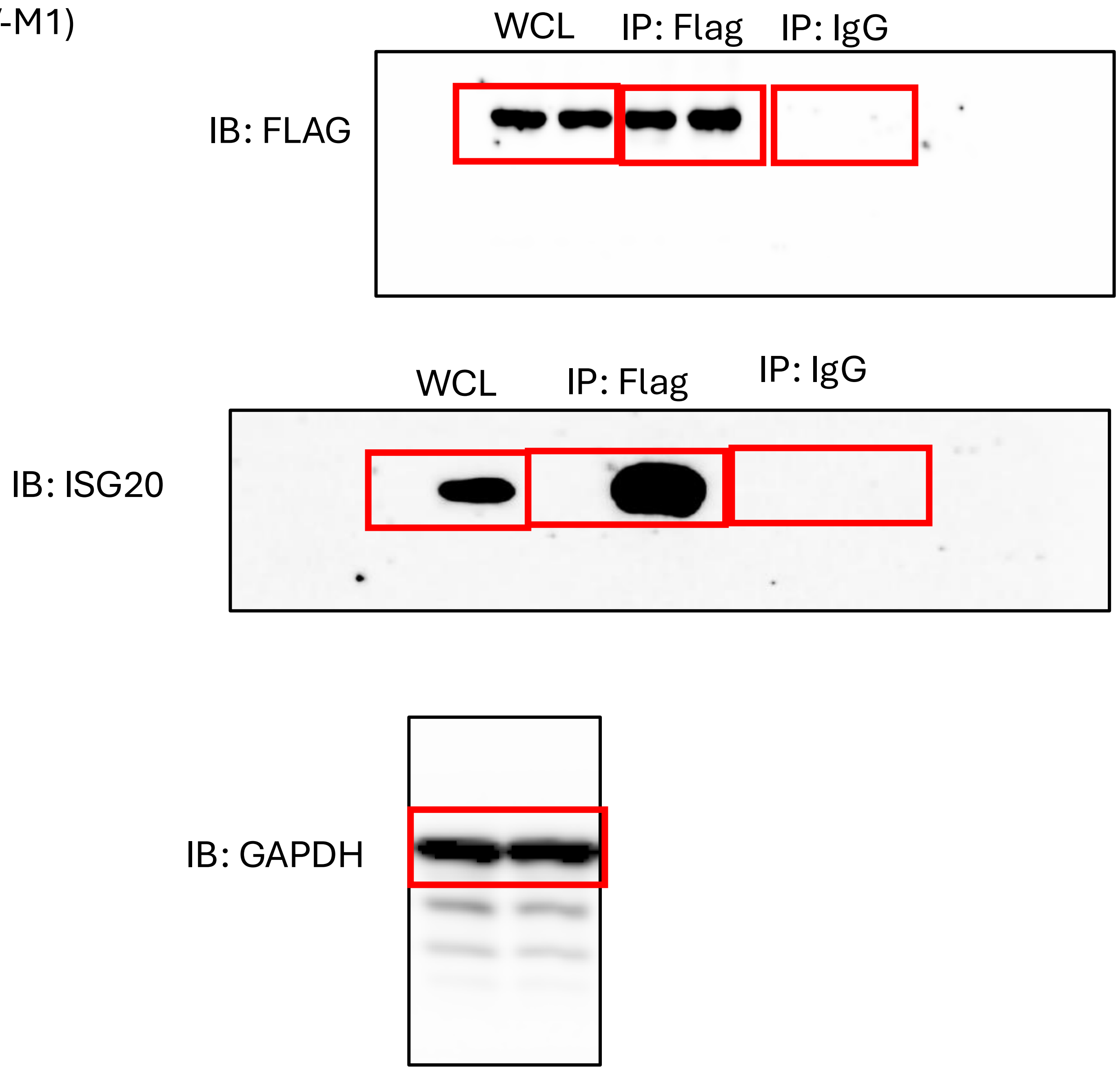

Fig 3 (3rd Panel: HBV-M2)

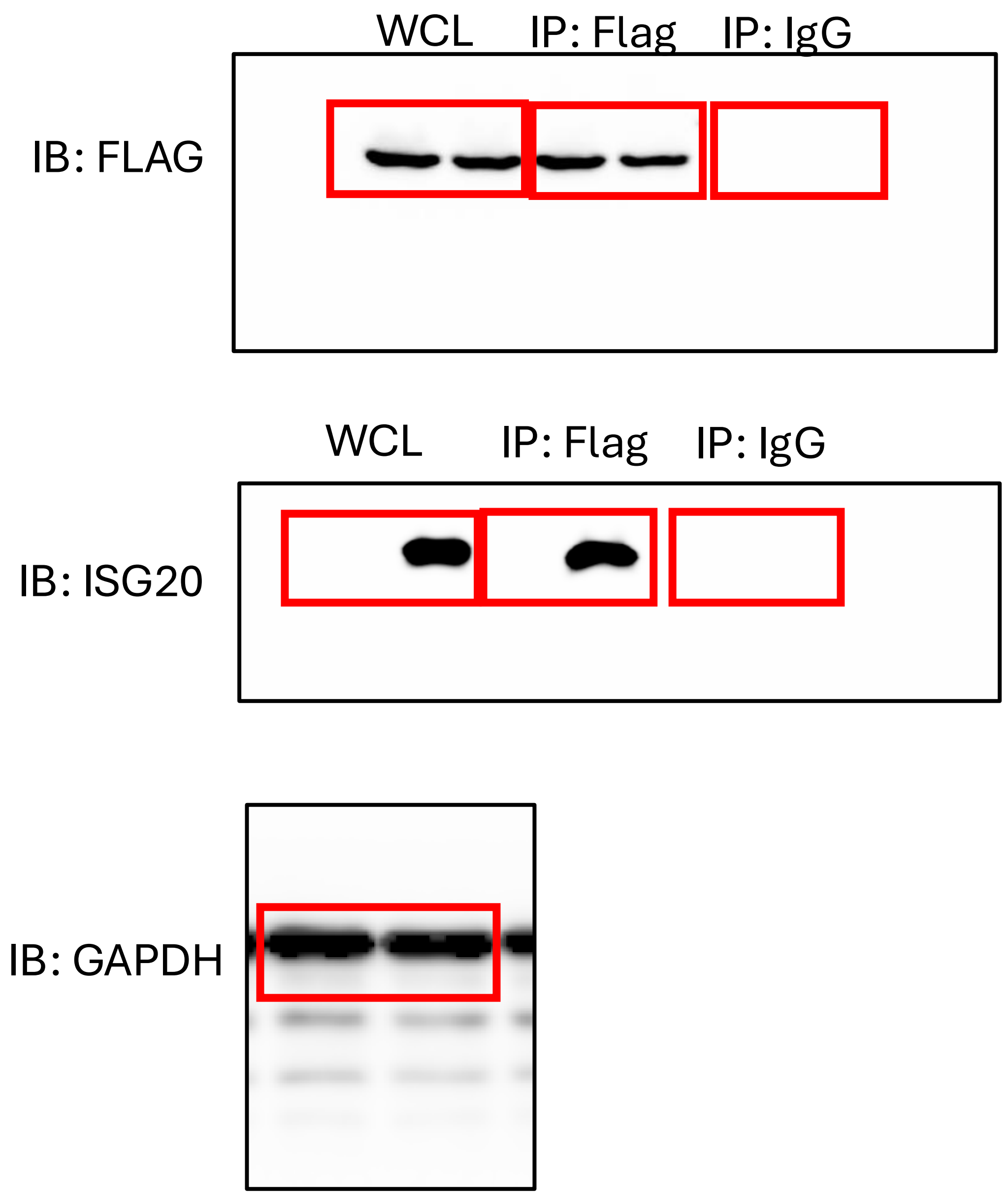

Fig 3 (4th Panel: HBV-M3)

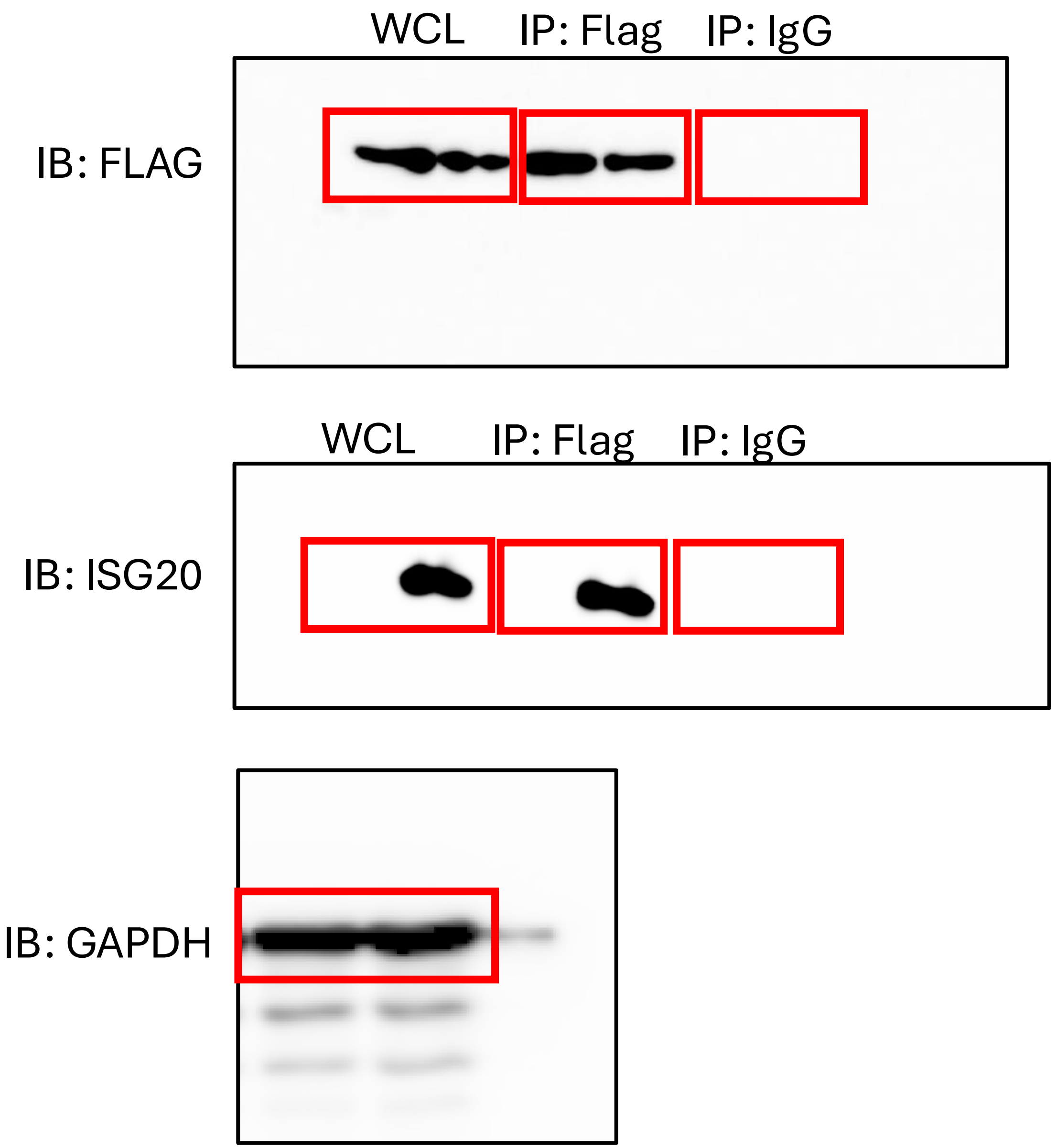

Fig 4A

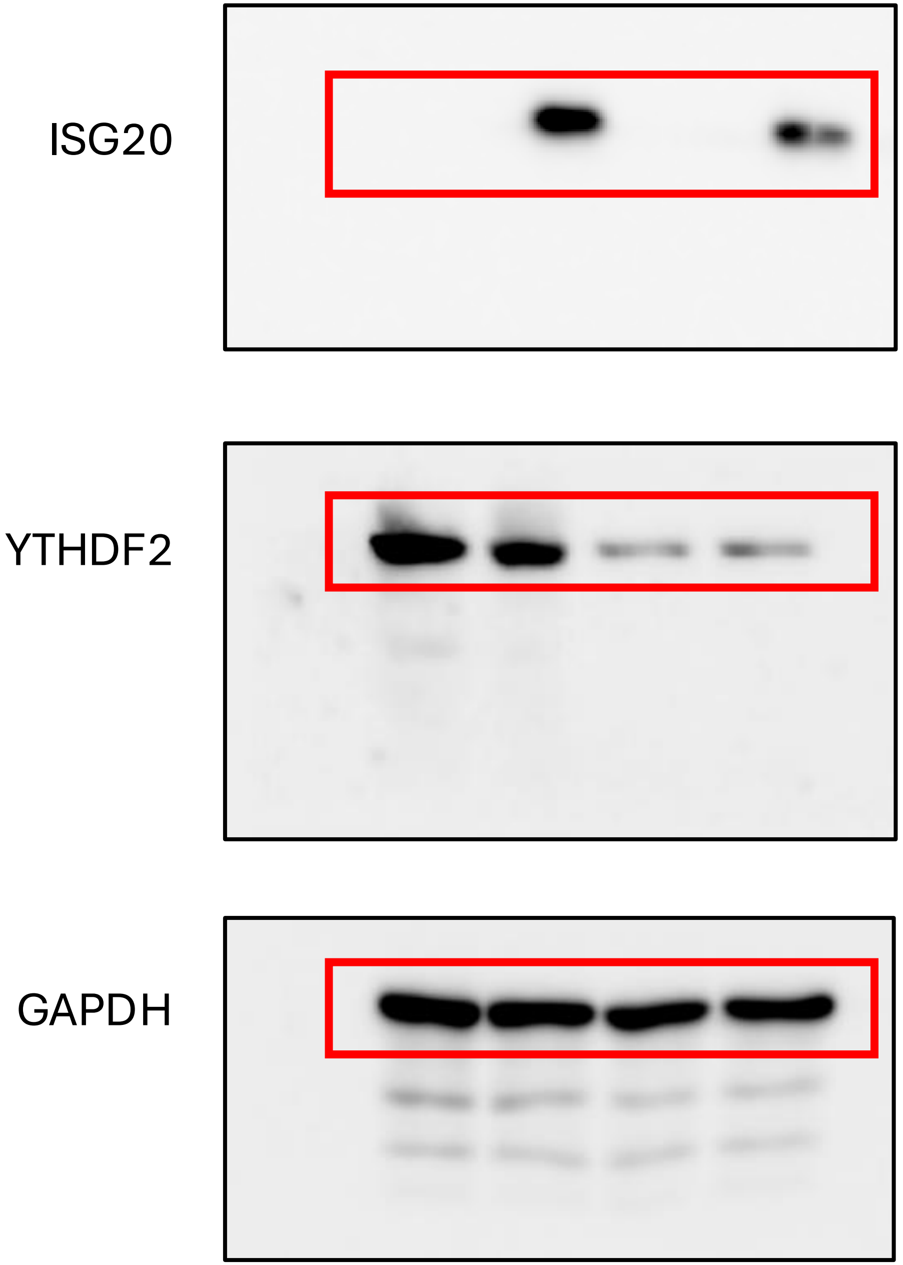

Fig 4B

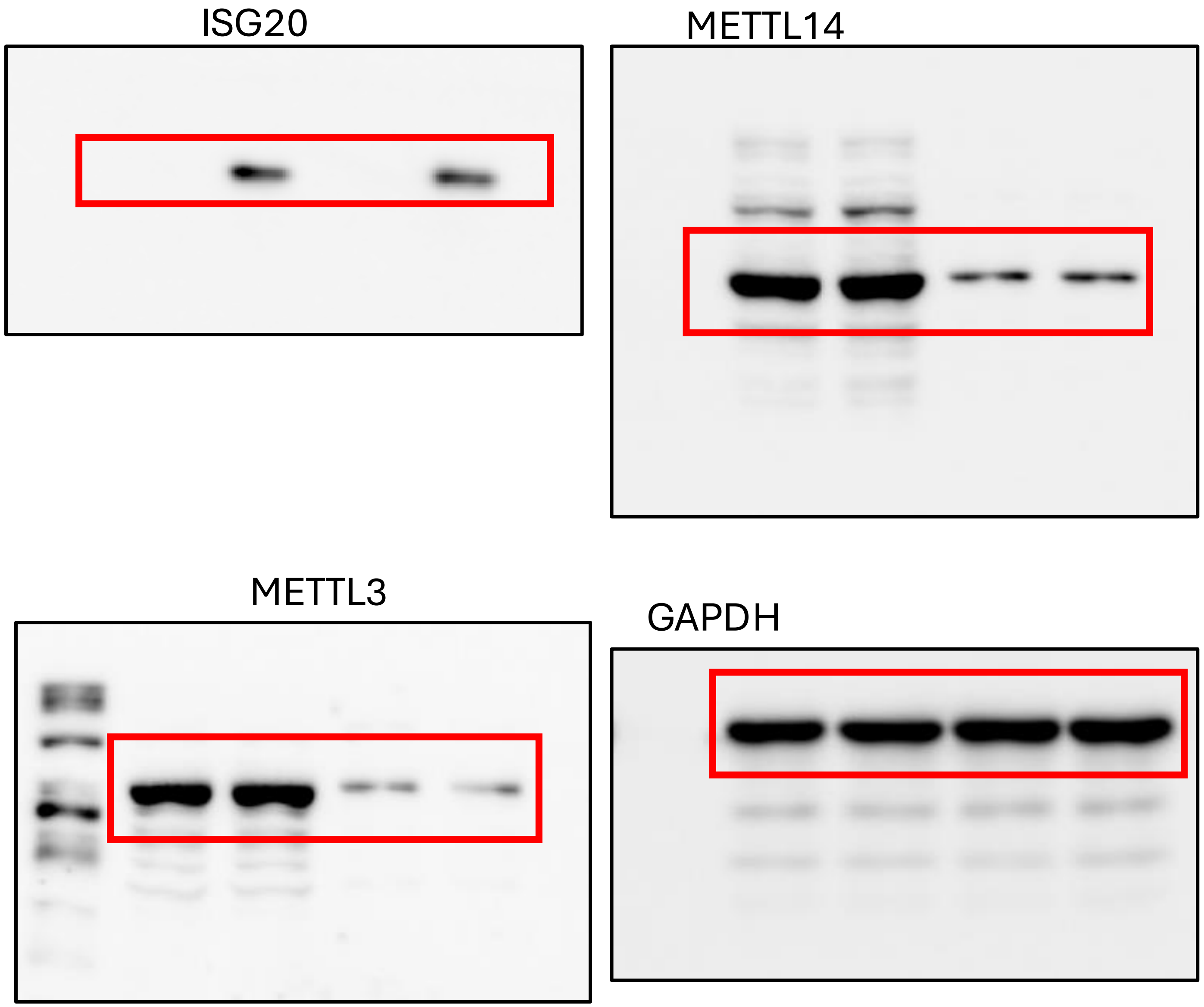

S2 Fig A (1st Panel: HBV-WT)

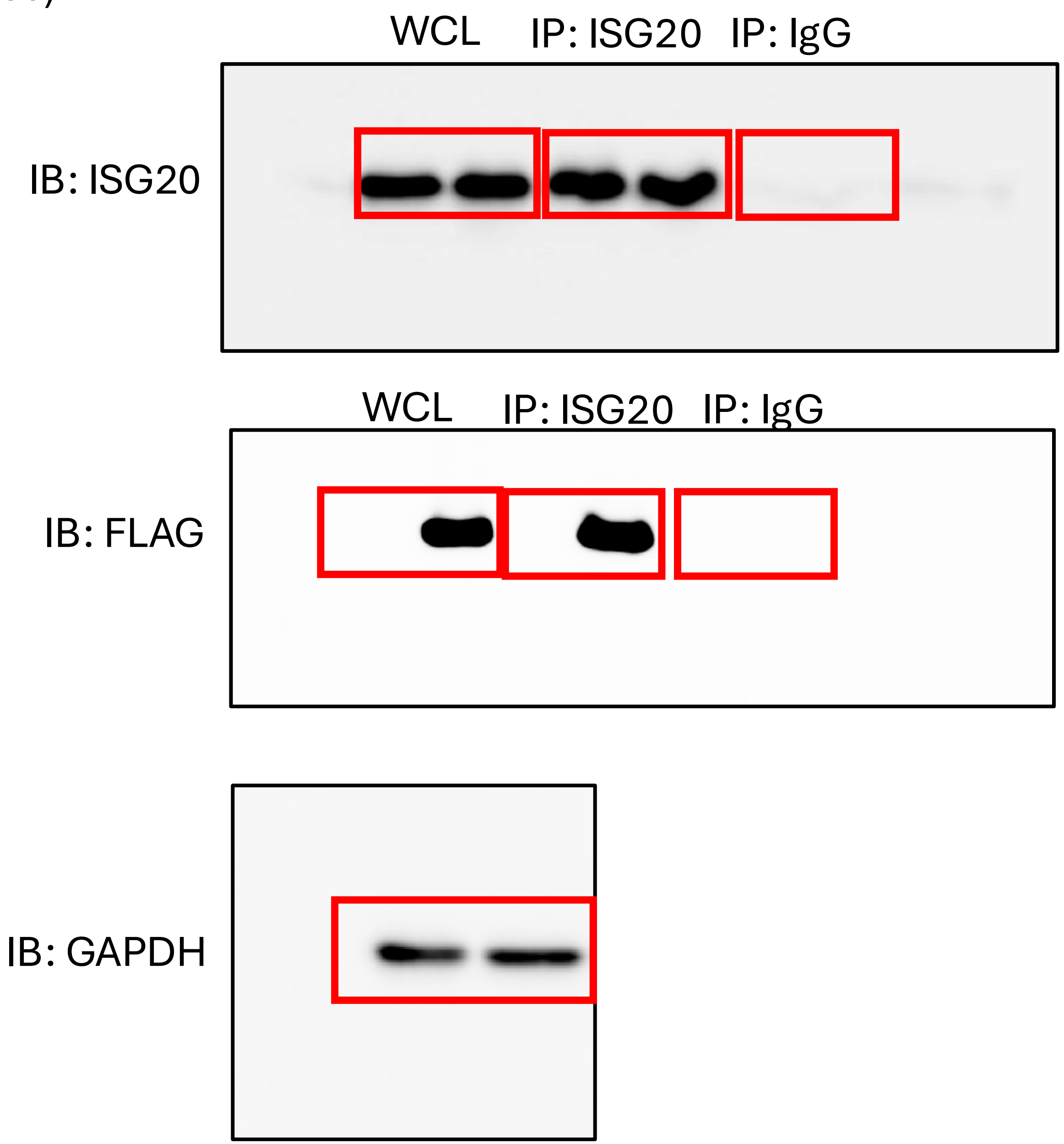

S2 Fig A (2nd Panel: HBV-M1)

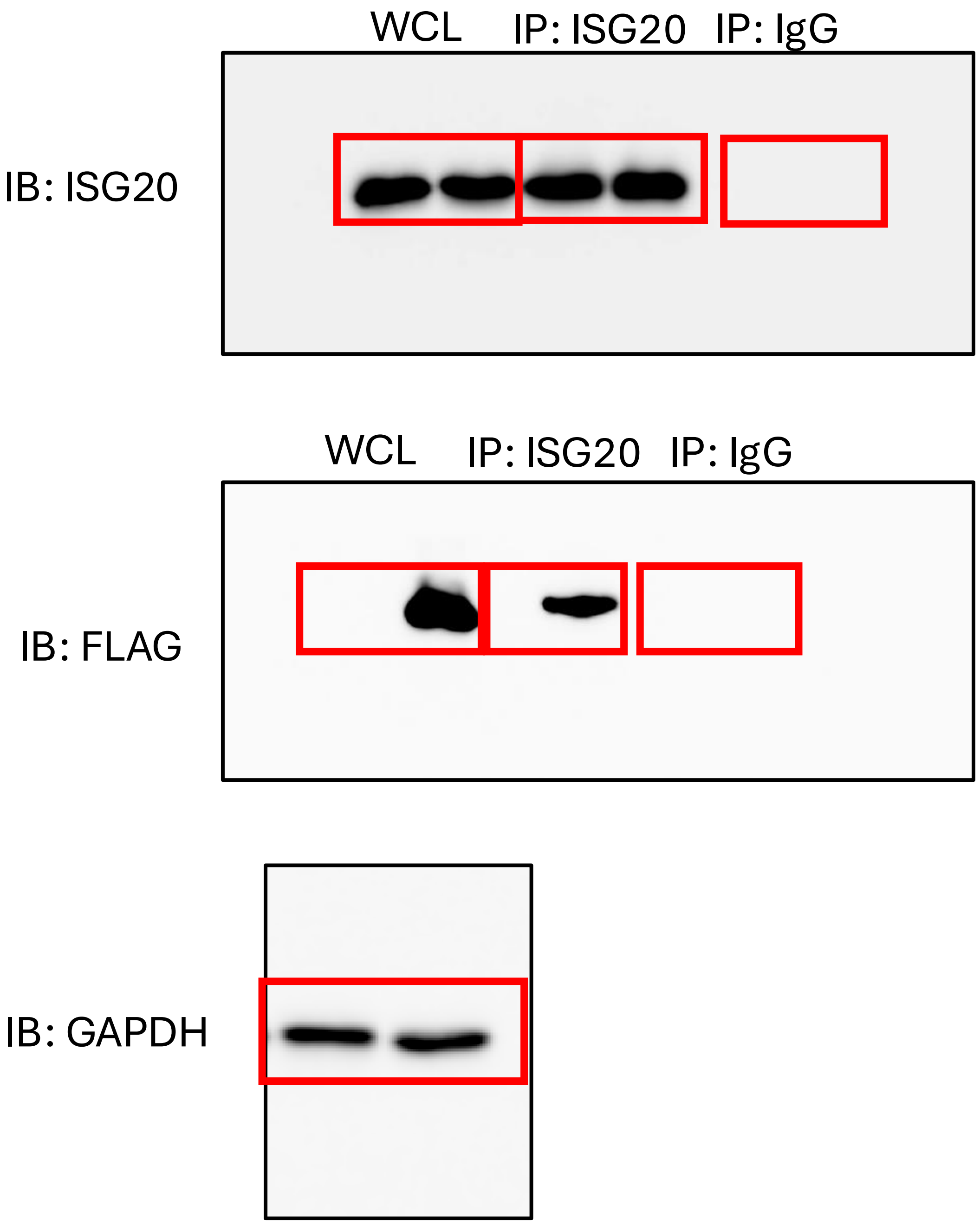

S2 Fig A (3rd Panel: HBV-M2)

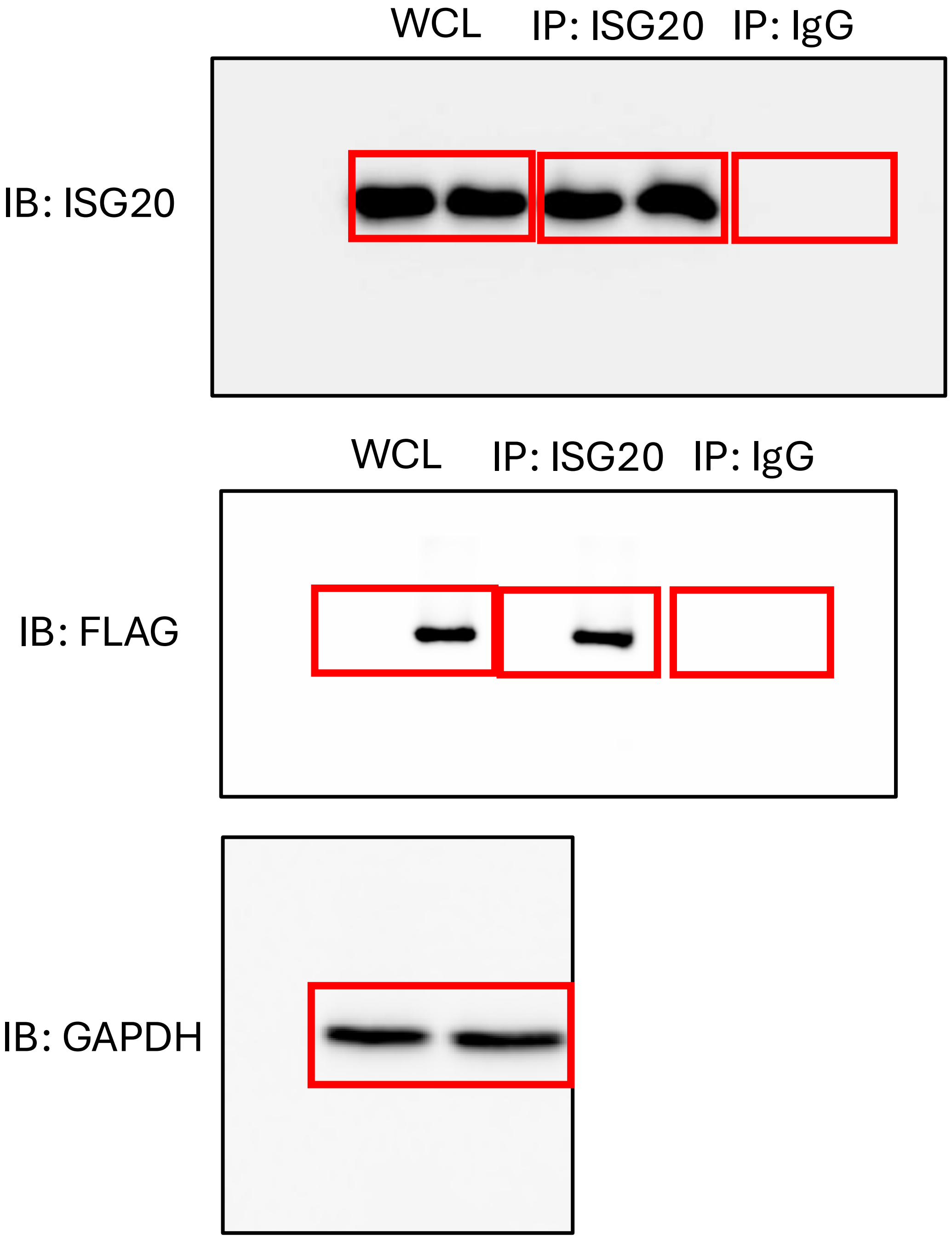

S2 Fig A (4th Panel: HBV-M3)

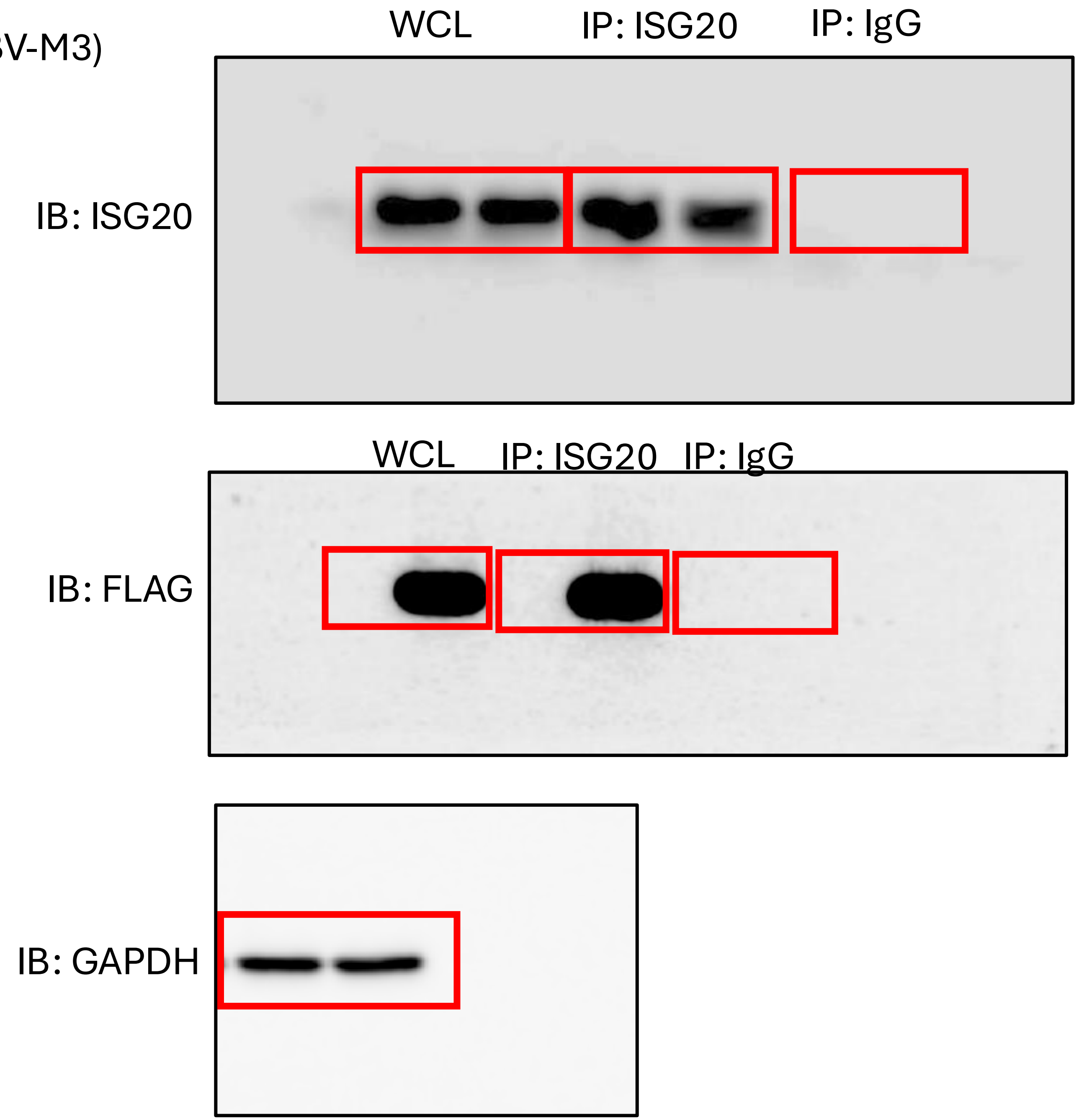

S2 Fig C (1st Panel: HBV-WT)

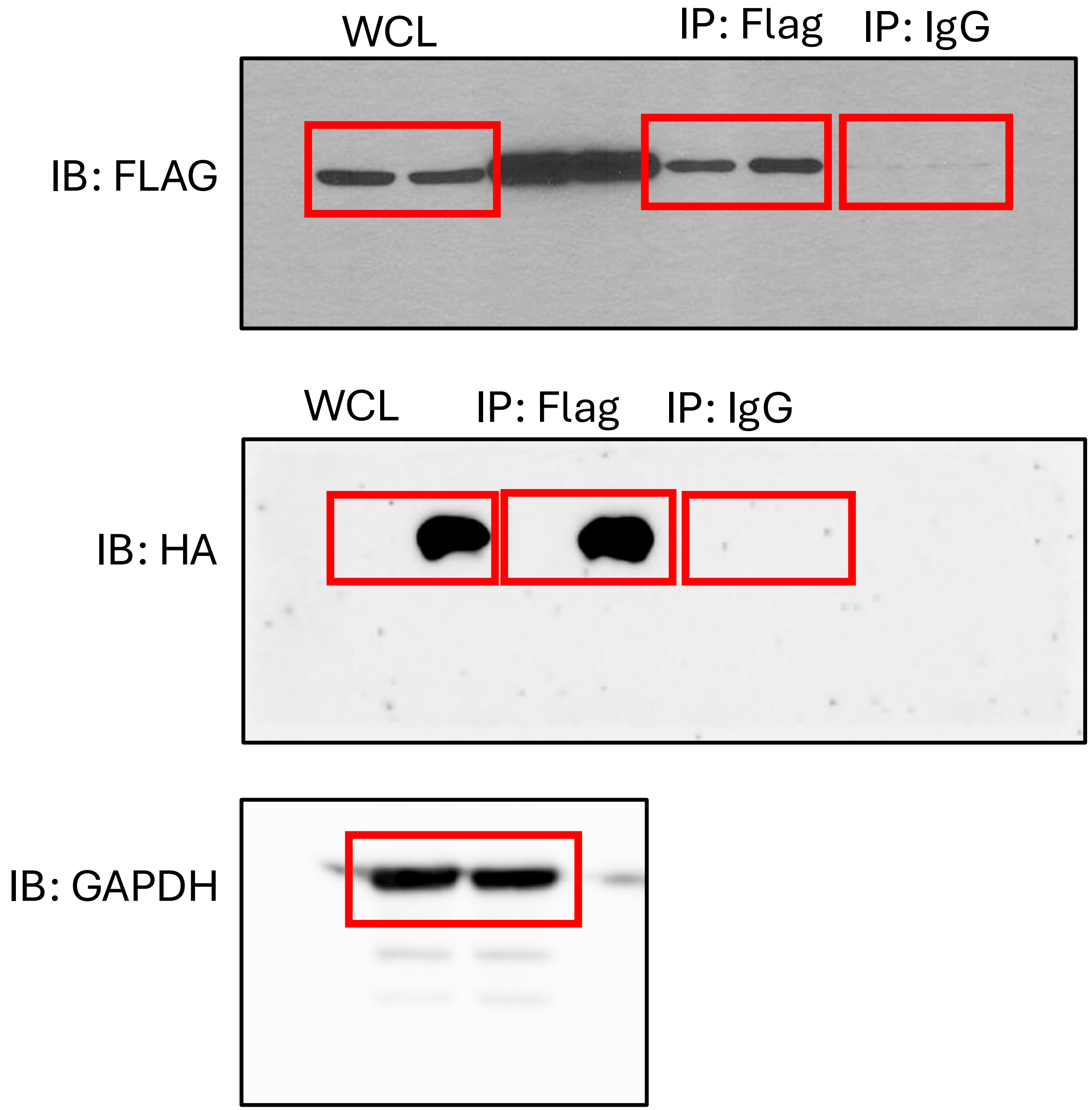

S2 Fig C (2nd Panel: HBV-M1)

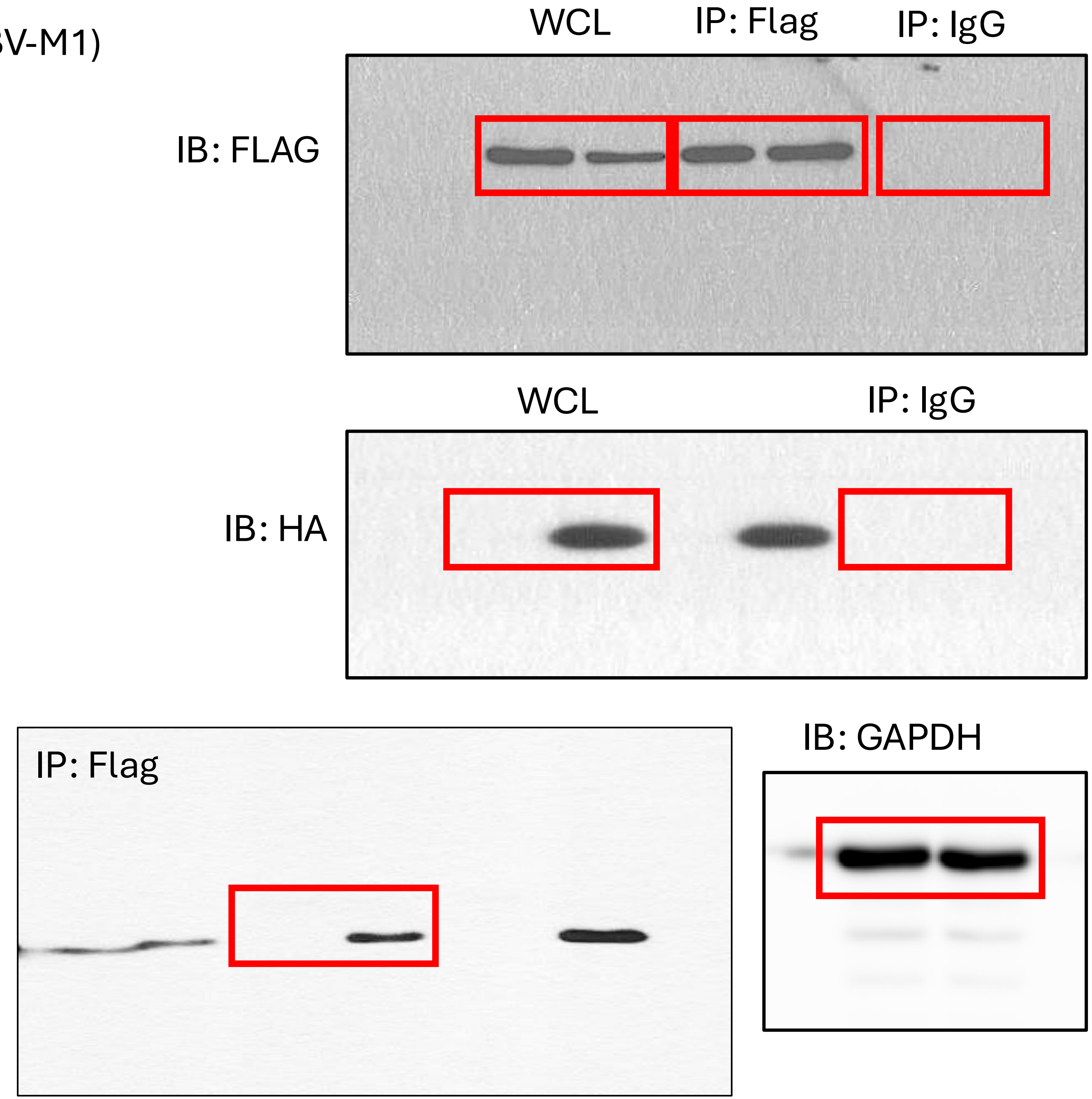

S2 Fig C (3rd Panel: HBV-M2)

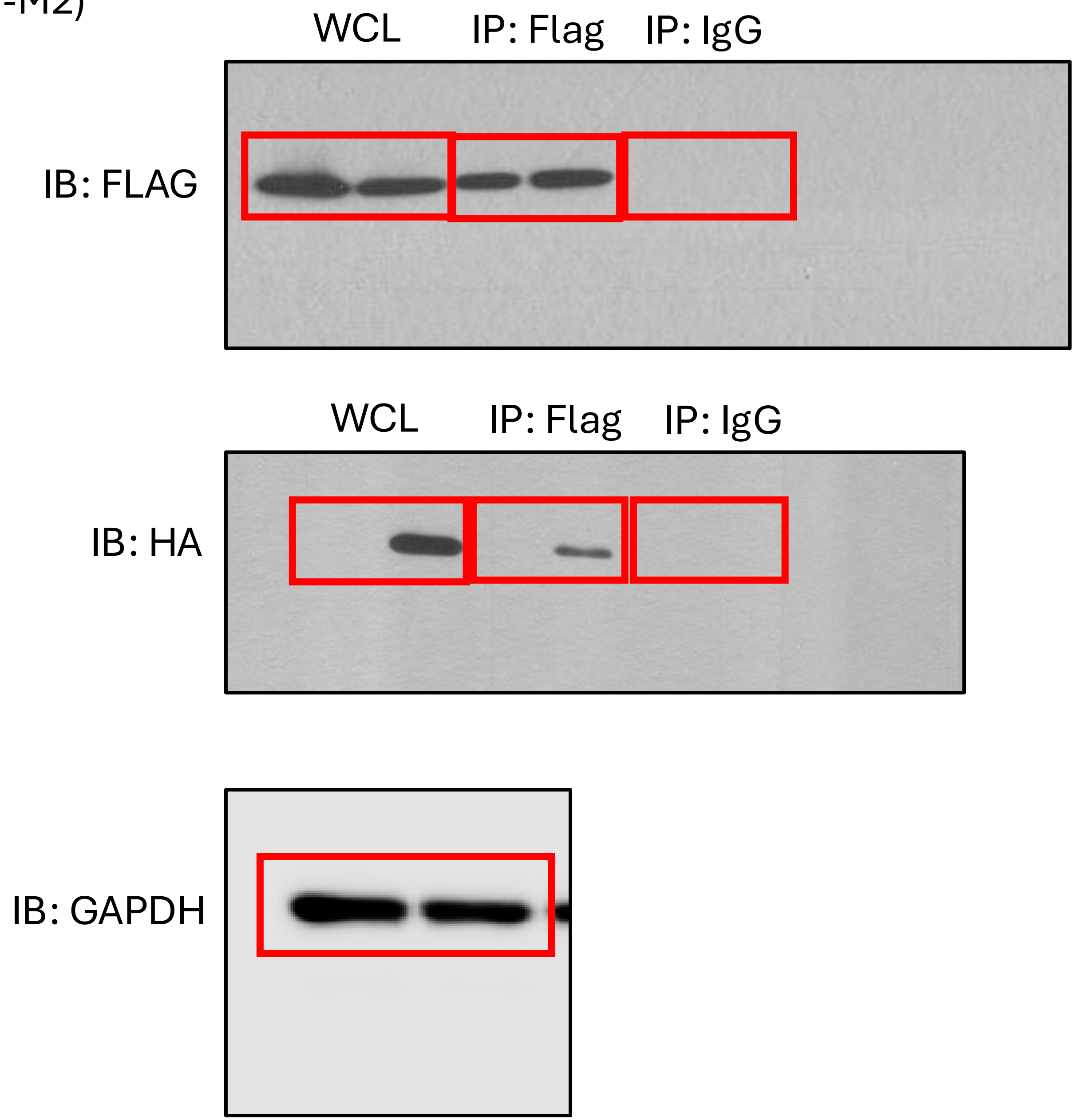

S2 Fig C (4th Panel: HBV-M3)

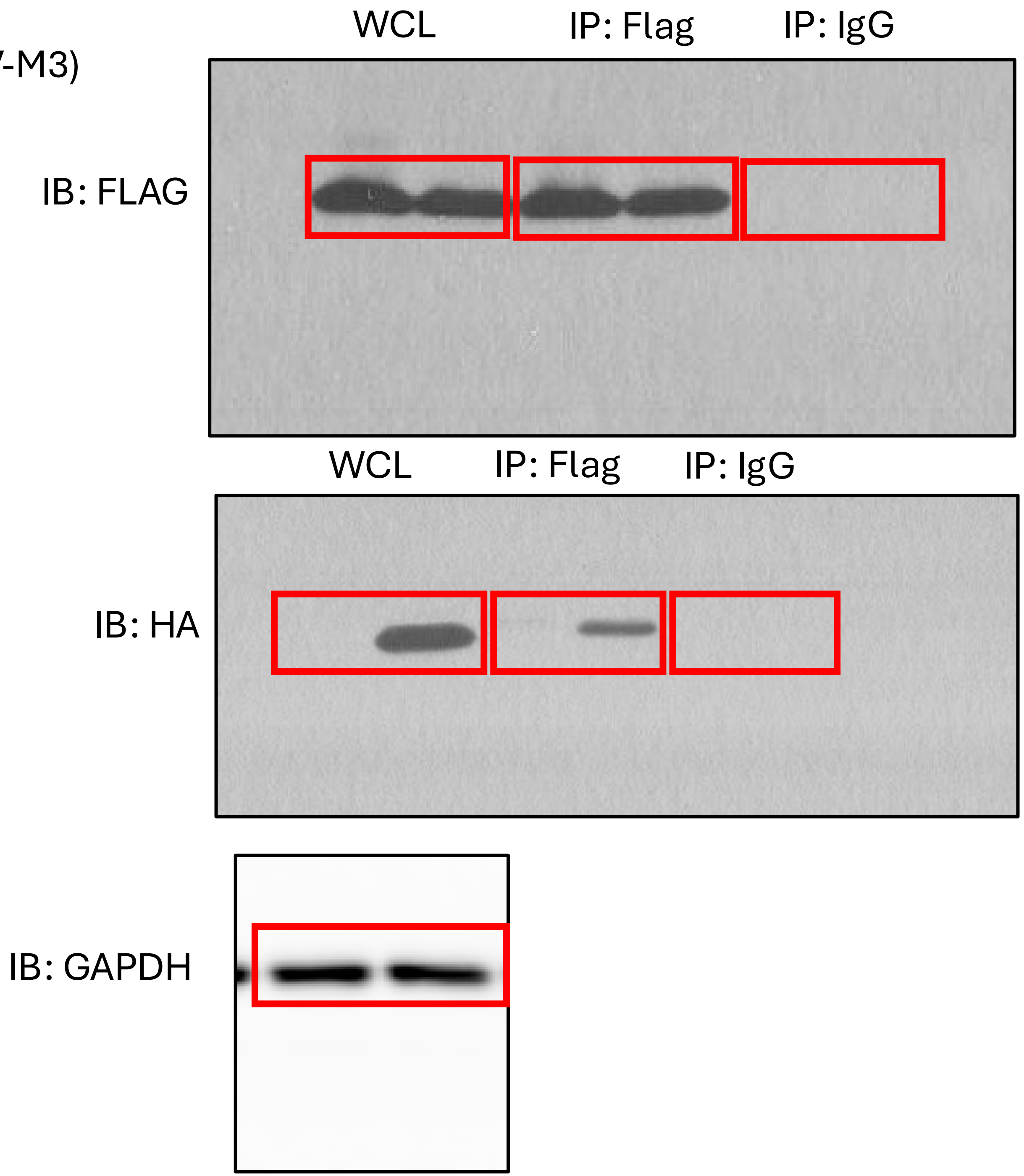

Supplement: S1 Data — (ZIP) [file ppat.1014336.s001.zip › Imam et al ISG20 Raw data/Raw Images.pdf]
